# versaFlow: a versatile pipeline for resolution adapted diffusion MRI processing and its application to studying the variability of the PRIME-DE database

**DOI:** 10.3389/fninf.2023.1191200

**Published:** 2023-08-10

**Authors:** Alex Valcourt Caron, Amir Shmuel, Ziqi Hao, Maxime Descoteaux

**Affiliations:** ^1^Sherbrooke Connectivity Imaging Laboratory, Computer Science Department, Université de Sherbrooke, Sherbrooke, QC, Canada; ^2^Brain Imaging Signals Lab, McConnell Brain Imaging Centre, Montreal Neurological Institute, McGill University, Montreal, QC, Canada

**Keywords:** non-human primates, magnetic resonance, diffusion, medical imaging, pipeline, high-performance computing, variability, reproducibility

## Abstract

The lack of “gold standards” in Diffusion Weighted Imaging (DWI) makes validation cumbersome. To tackle this task, studies use translational analysis where results in humans are benchmarked against findings in other species. Non-Human Primates (NHP) are particularly interesting for this, as their cytoarchitecture is closely related to humans. However, tools used for processing and analysis must be adapted and finely tuned to work well on NHP images. Here, we propose versaFlow, a modular pipeline implemented in Nextflow, designed for robustness and scalability. The pipeline is tailored to *in vivo* NHP DWI at any spatial resolution; it allows for maintainability and customization. Processes and workflows are implemented using cutting-edge and state-of-the-art Magnetic Resonance Imaging (MRI) processing technologies and diffusion modeling algorithms, namely Diffusion Tensor Imaging (DTI), Constrained Spherical Deconvolution (CSD), and DIstribution of Anisotropic MicrOstructural eNvironments in Diffusion-compartment imaging (DIAMOND). Using versaFlow, we provide an in-depth study of the variability of diffusion metrics computed on 32 subjects from 3 sites of the Primate Data Exchange (PRIME-DE), which contains anatomical T1-weighted (T1w) and T2-weighted (T2w) images, functional MRI (fMRI), and DWI of NHP brains. This dataset includes images acquired over a range of resolutions, using single and multi-shell gradient samplings, on multiple scanner vendors. We perform a reproducibility study of the processing of versaFlow using the Aix-Marseilles site's data, to ensure that our implementation has minimal impact on the variability observed in subsequent analyses. We report very high reproducibility for the majority of metrics; only gamma distribution parameters of DIAMOND display less reproducible behaviors, due to the absence of a mechanism to enforce a random number seed in the software we used. This should be taken into consideration when future applications are performed. We show that the PRIME-DE diffusion data exhibits a great level of variability, similar or greater than results obtained in human studies. Its usage should be done carefully to prevent instilling uncertainty in statistical analyses. This hints at a need for sufficient harmonization in acquisition protocols and for the development of robust algorithms capable of managing the variability induced in imaging due to differences in scanner models and/or vendors.

## 1. Introduction

*Magnetic Resonance Imaging* (MRI) is a modality of medical imaging that has seen an exponential increase in interest in the past few decades. It is the only non-invasive technology able to acquire both functional and structural information from soft tissues, which makes it the preferential choice for the study of brain functions and connectivity with the aim, notably, of mapping the human connectome. For that matter, *Diffusion Weighted Imaging* (DWI), enabling the introspection of the diffusion process of water molecules both inside and around the axonal structure, is the favored MRI modality. However, the study of *Diffusion Weighted* (DW) images is riddled with challenges that the scientific community has yet to overcome (Jones, 2010a; Jones et al., 2013; O'Donnell and Pasternak, 2015), especially when it comes to tractography (Maier-Hein et al., 2017; Schilling et al., 2017, 2021b; Calamante, 2019; Rheault et al., 2020), and even more in ensemble studies using multiple sites and multiple scanner vendors (De Santis et al., 2019; Andica et al., 2020; Schilling et al., 2021a). Furthermore, the current equipment available still fails to provide gradients and slew rates with performance sufficient for high-resolution imaging at a decent *Signal-to-Noise Ratio* (SNR); the quality is restricted by eddy currents and phase-related distortions, to name a few. Still, all MRI-related techniques are limited since the best achievable imaging resolution remains at the macroscopic millimeter resolution, which is orders of magnitude larger than the sizes of the structures of interest, in the micrometer to the nanometer range. This results in partial volume effects (Alexander et al., 2001) at the interface between tissue structures. Those effects can be mitigated by the use of pulse sequences specifically tailored for sub-millimeter imaging (Tounekti et al., 2018; Grier et al., 2020), at the trade of increasing acquisition time. Nevertheless, lowering the size of acquired voxels is often not a viable option: it leads to a decrease in signal contributions induced by shrinking the proton pool size, which lowers SNR in the acquired images, and the longer times their acquisition requires results in higher probabilities of motion-related artifacts. Selecting a good set of acquisition parameters is thus a multi-objective optimization problem, a trade-off between the image resolution required for subsequent modeling and the acceptable level of noise, the duration of the imaging sequence and the type of subject, and a research setting vs. a clinical application, among others.

In DWI, this problem cannot be tackled directly, since there is no proper *gold standard* upon which to compare and rank imaging results obtained. Consequently, validation of study design and methods usually defer to stability or correlation analyses using multi-subject, longitudinal, or translational approaches. Comparing MRI acquisitions to tracer studies is known to provide good benchmarks in white matter, but can lack in specificity (Heilingoetter and Jensen, 2016), and its application involves the injection of invasive substances in the tissue of interest, requiring the subject to be sacrificed thereafter or the procedure to be performed *ex vivo*. Recently, multi-resolution frameworks have gained popularity, combining low-resolution DWI with high-resolution microscopy to refine the angular representation of diffusion (Howard et al., 2019). However, this technique is also limited to *ex vivo* studies, since microscopy requires slicing and staining of the brain tissues. Another technique that has made its mark is comparison to results in small animals. They enable the use of specialized equipment that operates at higher magnetic fields, advanced pulse sequences, as well as improved techniques to monitor the sources of subject's motion enabling imaging at finer spatial and angular resolutions while retaining good SNR. These methods are either inaccessible or ethically unacceptable in human studies.

Thanks to a few decades of research in human brain connectomics and large-scale endeavors like the *Human Connectome Project* (HCP) (Van Essen et al., 2012), for financing the collection of thousands of DW images on a large number of subjects from all around the world (Van Essen et al., 2012; Fan et al., 2016; Mazoyer et al., 2016; Mansour et al., 2023), efficient acquisition protocols, practices, and guidelines were created for human studies (Wedeen et al., 2008; Moeller et al., 2010; Tournier et al., 2011; Caruyer et al., 2013; Jones et al., 2013; Sotiropoulos et al., 2013), as well new more sensitive and precise equipment (Setsompop et al., 2013; Nowogrodzki, 2018; Quettier et al., 2020). This has allowed the community to generate a corpus of algorithms and software tailored to efficiently and robustly process DW images acquired from human brains (Smith et al., 2004; Avants et al., 2008; Garyfallidis et al., 2014; Tournier et al., 2019), and put in place automatized processing pipelines (Pierpaoli et al., 2010; Gorgolewski et al., 2011; Autio et al., 2020; Theaud et al., 2020; Cieslak et al., 2021). *In vivo Non-Human Primate* (NHP) studies have not seen this kind of attention yet; cohorts of subjects are usually small and the lack of standardized setups across sites makes it difficult to provide and furthermore homogenize protocols between them. Nonetheless, it is still required to adequately preprocess those images before fitting models and carrying out data analysis. Algorithms used to that extent must be fine-tuned to NHP-specific requirements. While re-using pipelines developed for humans on NHP imaging is a possibility, most of them do not offer the options for extensive re-configuration required for applying them to NHP high-resolution DWI. Moreover, most of them are distributed as black-boxes, difficult to customize or extend, which makes adapting them to different studies time-consuming.

The pipeline we present in this study alleviates most if not all of those problems. Using Nextflow (Tommaso et al., 2017) and the latest implementation of its DSL2 framework, we designed a collection of processing modules consisting of pipeline processes and workflows, each targeting a specific set of preprocessing, model reconstruction, quantity measurement, or utility algorithms, using cutting edge DWI processing technologies such as FSL (Smith et al., 2004), ANTs (Avants et al., 2008), Dipy (Garyfallidis et al., 2014), and Mrtrix (Tournier et al., 2019). We then created *versaFlow* using those modules, a pipeline that self-adapts given its input's spatial and angular resolutions, guaranteeing optimal algorithm's configurations at execution. The use of Nextflow ensures its scalability on a wide range of computing infrastructures, from a local computer to multiple nodes in a *High-Performance Computing* (HPC) facility. Container technologies (Docker and Singularity; Kurtzer et al., 2017) allow for the encapsulation of all dependencies and their versioning. This combination makes a robust and automated execution possible while keeping installation and maintenance as simple as possible for the end user.

To test the pipeline efficiency on *in vivo* NHP DWI data, we processed 32 datasets from 3 sites of the *Primate Data Exchange* (PRIME-DE) database (Milham et al., 2018; Neff, 2019). Together, they provide diffusion-weighted images acquired *in vivo* using human scanners from multiple vendors. They feature multiple spatial and angular resolutions, single-shell and multi-shell b-value samplings, and artifacts of different ranges of magnitudes depending on the site where the acquisition was performed. We provide a brief reproducibility study of the pipeline results, as well as an in-depth variability study of diffusion modeling and measurements. We show that just as reported in similar human studies, the *in vivo* NHP DWI data provided in the PRIME-DE exhibits a high level of variability. The various metrics produced by *versaFlow* show a clear increase in the variability when pooling together data from multiple sites, acquired using equipment manufactured by different vendors, as well as different models from the same vendors. To pursue adequate quantitative analysis, users of the data should take great care and preferably apply a harmonization technique before carrying out analysis.

## 2. Method

### 2.1. Processing library

Three core concepts were taken into account for the development of the processing library:

- Efficiency

High-resolution images (sub-millimeter spatial resolution and dense gradient sampling at multiple b-value points) are heavy (hundreds of megabytes to a few gigabytes), in particular for DW images, where a complete MRI volume is acquired for each gradient direction. Combining high spatial and angular resolution leads to image files of multiple gigabytes and a number of voxels on the order of several hundreds of millions. Processing must thus be efficient and parallelized as much as possible. To achieve such a degree of efficiency, we coded the pipeline structure using Nextflow (Tommaso et al., 2017), a meta-scheduling language enabling automatic optimization and execution of a processing tree over multiple datasets and multiple processing nodes. We also took great care in selecting optimized and parallelized implementations of the algorithms we integrated into our processing chain.

- Modularity and morphability

State-of-the-art methods in DWI are still changing rapidly. For a good pipeline to stay relevant, it needs to have the capacity to morph to new standards and integrate novel techniques. Using the DSL2 framework of Nextflow, we created a highly modular library of processes and workflows, used as building blocks in our pipeline. This allowed for a high-level architecture in the final coded pipeline, with a finer description of the dataflows encapsulated in modules referring to specific processing steps. Using this paradigm makes it easier for new pipelines to be developed that only change a subset of processing steps or add new workflows on top of older ones.

- Usability and reproducibility

The libraries used to process DW images are ever-changing. It is thus impossible to guarantee a robust execution of our pipeline on versions of its dependencies released in the future. Docker and Singularity (Kurtzer et al., 2017)—two technologies enabling the encapsulation of libraries in portable packages—were used to wrap up the numerous applications called in the different processing steps of the pipeline. They enable locking the versions of the dependencies into a pre-packaged image and remove the need for the end-user to install and manage them. In addition, they allow for multi-stage building of images, which makes it easy to add new dependencies and modify a set of versions in the case of an update of the underlying pipeline or for the needs of a specific study.

**Figure 1:**
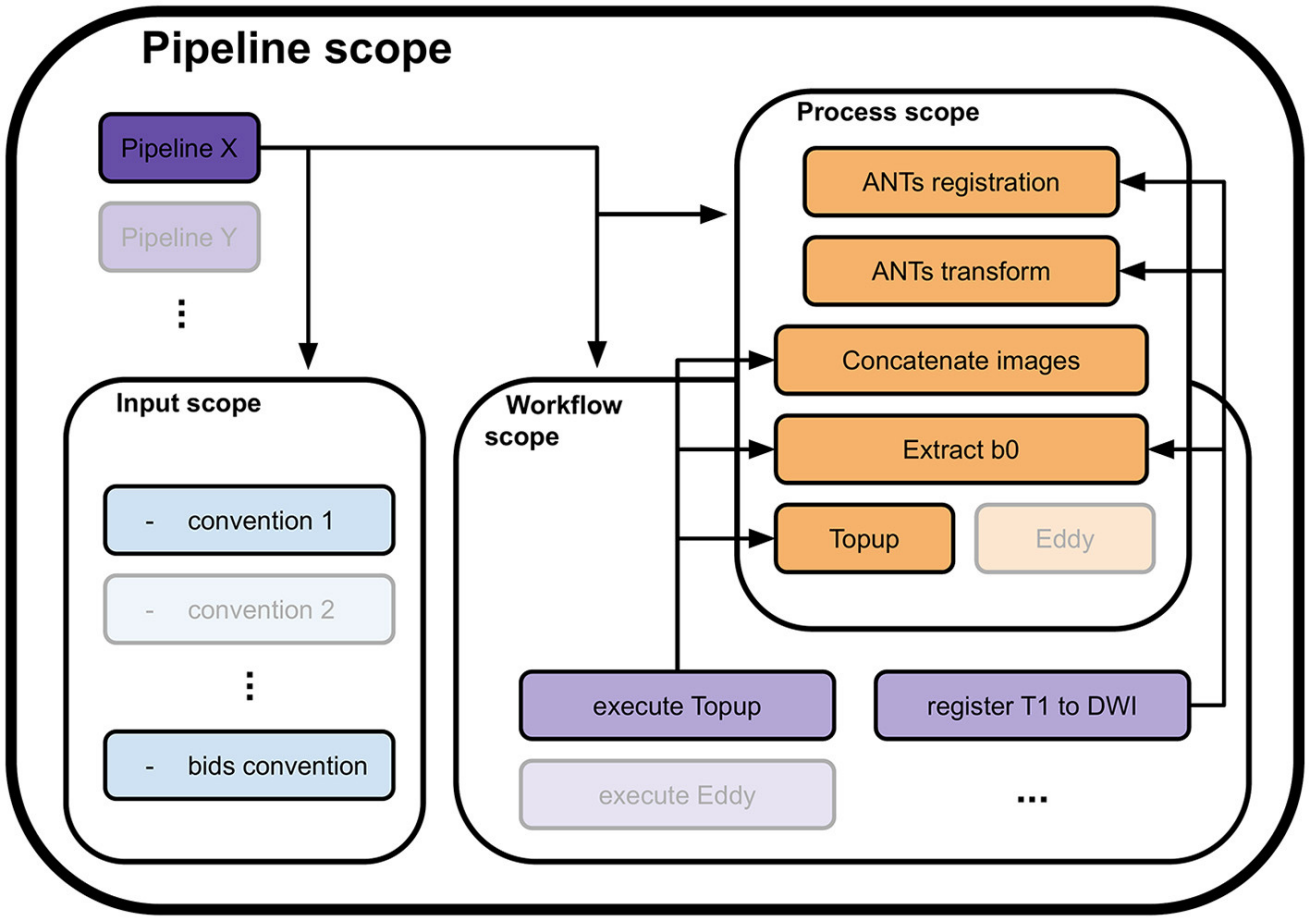
Four scopes composing the Nextflow library and how they interface with each other. A pipeline definition (Pipeline X for example) is placed at the root of the library. To load the data, it uses one or more conventions from the input scope (convention 1 and the bids convention). Then, to process it, it calls utilities in either or both the process scope (ANTs registration, Concatenate images, and others) and the workflow scope (execute *Topup*, then register T1w to DWI). A call to a utility in the workflow scope will trigger calls to processes in the process scope they include to process the data (execute *Topup* firsts calls a concatenation of two opposite phase encoding DW images and extracts their b0 volumes, to pass the result to *Topup* for phase distortions correction). The input, workflow, and process scopes form the core of the Nextflow library. Their content can be used by any pipeline defined in the pipeline scope, allowing high levels of code reusability.

To address these concepts, the library was fragmented into 4 nested and interlocked scopes: pipeline scope, input scope, process scope, and workflow scope, as depicted in Figure 1. Of them, only the process scope contains the actual calls to algorithms and their input requirements. It encapsulates the actual workhorse of the pipeline and forms the processes module.

All other scopes use Nextflow workflow objects and only specify the flow of data between processes and other workflows they include. The input scope gathers pieces of code necessary to translate the internal representation in disk memory of the input data into a data structure that is digestible by a pipeline. When included, it enforces the input convention of its pipeline. It can be changed to fit a specific study or project. The pipeline scope contains all complete pipelines. A valid pipeline uses one or more input conventions from the input scope and describes as minimally as possible the flow of data between processes and sub-workflows it includes. All data flow descriptions that can potentially be reused are transferred to the workflow scope. This scope contains sub-parts of the processing that together form a coherent ensemble, yet cannot be efficiently specified as a single process. The workflows in this scope use processes and other workflows as building blocks and define the passage of data between them to form a processing sequence. An example of such a workflow would be the execution of *Topup* (a light purple workflow in Figure 1), including data preparation, which executes the following processes sequentially: selection of b0 volumes from the different phase encoded DW images, concatenation of b0 volumes, computation of the susceptibility field, and application of the field to the DW images.

#### 2.1.1. Code availability

*versaFlow* and the Nextflow processing library are available on github at github.com/AlexVCaron/versaFlow. Docker images containing all dependencies and requirements to run *versaFlow* are located at hub.docker.com/r/avcaron/versa. The python code enabling custom configuration of several algorithms in the pipeline and supplying the Docker and Singularity build systems is available at github.com/AlexVCaron/mrHARDI.

#### 2.1.2. Data input

In addition to the DWI volumes and their associated bval/bvec files describing the gradient sampling, the pipeline requires as input a T1w anatomical image. The user can also supply masks in either or both diffusion and anatomical space, as well as partial volume maps of *White Matter* (WM), *Gray Matter* (GM), and *Cerebrospinal Fluid* (CSF), in anatomical space. Reverse phase acquired diffusion images can be provided, as either a single b0 volume, multiple b0 organized as a 4D volume, or a full DWI 4D volume with bval/bvec files. For some of the processing steps, the pipeline also requires the input of some metadata associated with the acquisition of the DWI and reverse-phase encoded volumes. Metadata parameters that are consistent across all images can be supplied using the Nextflow configuration file. In case of varying parameters between the images, a json file must be provided alongside both the forward and reverse-phase encoded volumes.

#### 2.1.3. Processing steps

**Figure 2:**
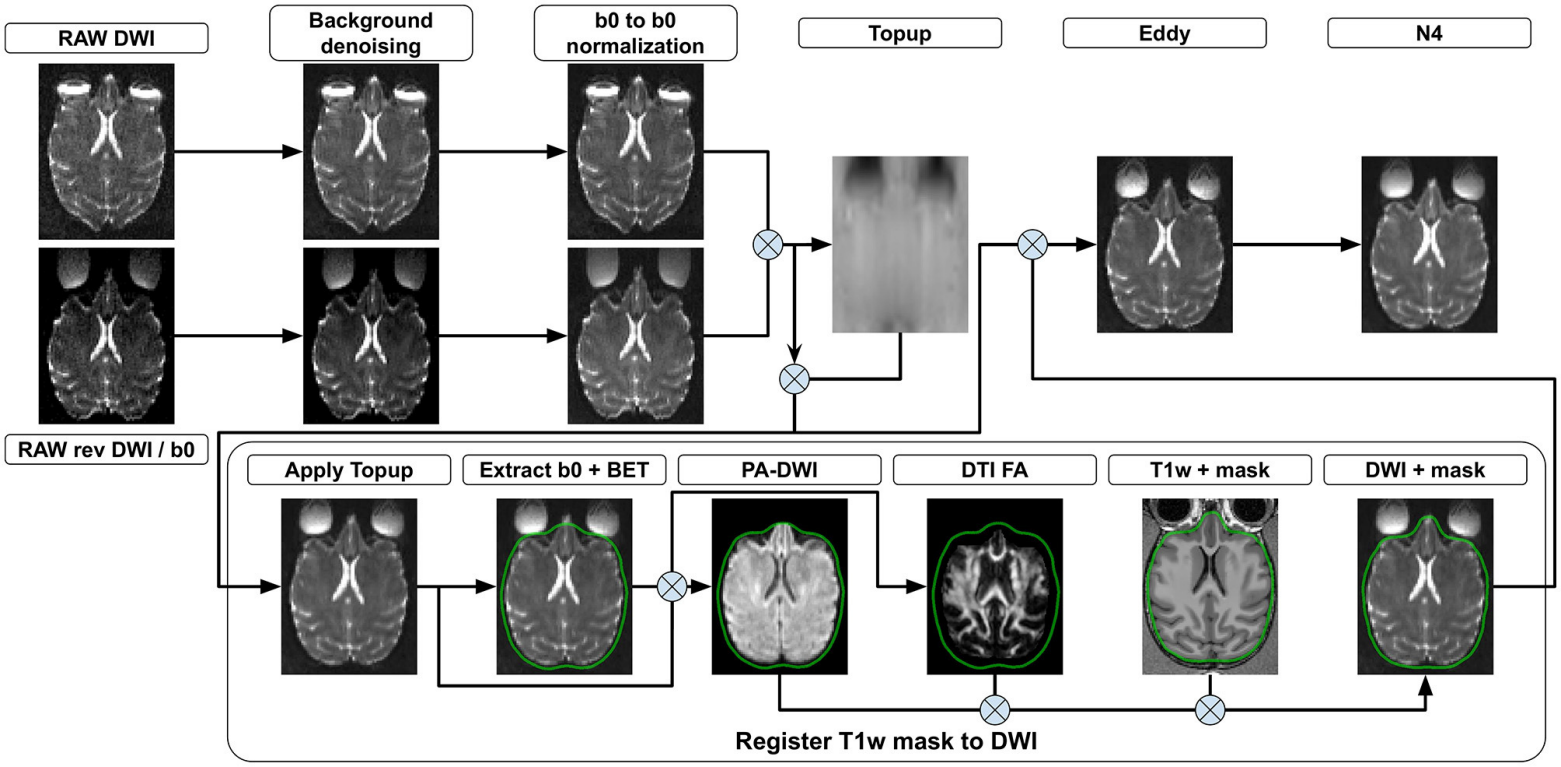
DWI preprocessing steps. (Background denoising) Input 4D DW images from both phase encoding directions are denoised to remove background noise. (b0 to b0 normalization) The signal is normalized such that all b0 volumes in both phase encoding directions exhibit the same mean value. Each 3D diffusion volume is normalized using as a reference either the b0 volume coming before it, after it or a linear interpolation of both. (Topup) b0 volumes from both phase encoding directions are concatenated and a deformation field is estimated. (Apply Topup) This field is applied on the forward phase encoded DW image to produce an undistorted 4D DW image. (Extract b0 + BET) A b0 volume is extracted from the undistorted image and used to compute a brain mask. All voxels outside the brain mask are set to 0 in the mean b0 volume and the undistorted 4D DW image. (PA DWI) The masked undistorted 4D DW image is used to compute a powder-averaged DWI image and (DTI FA) a fractional anisotropy map. (Register T1w to DWI) A transformation is computed by registering the T1w volume to both the mean b0 and the FA volumes. The brain mask in the T1w image space is then transformed into the DW image space using the computed transformation. (Eddy) Motion and eddy currents corrections are computed using the deformation field from *Topup*, the brain mask, and the 4D DW image from both phase encoding directions. (N4) The 4D DW image is corrected for intensity distortions.

**Figure 3:**
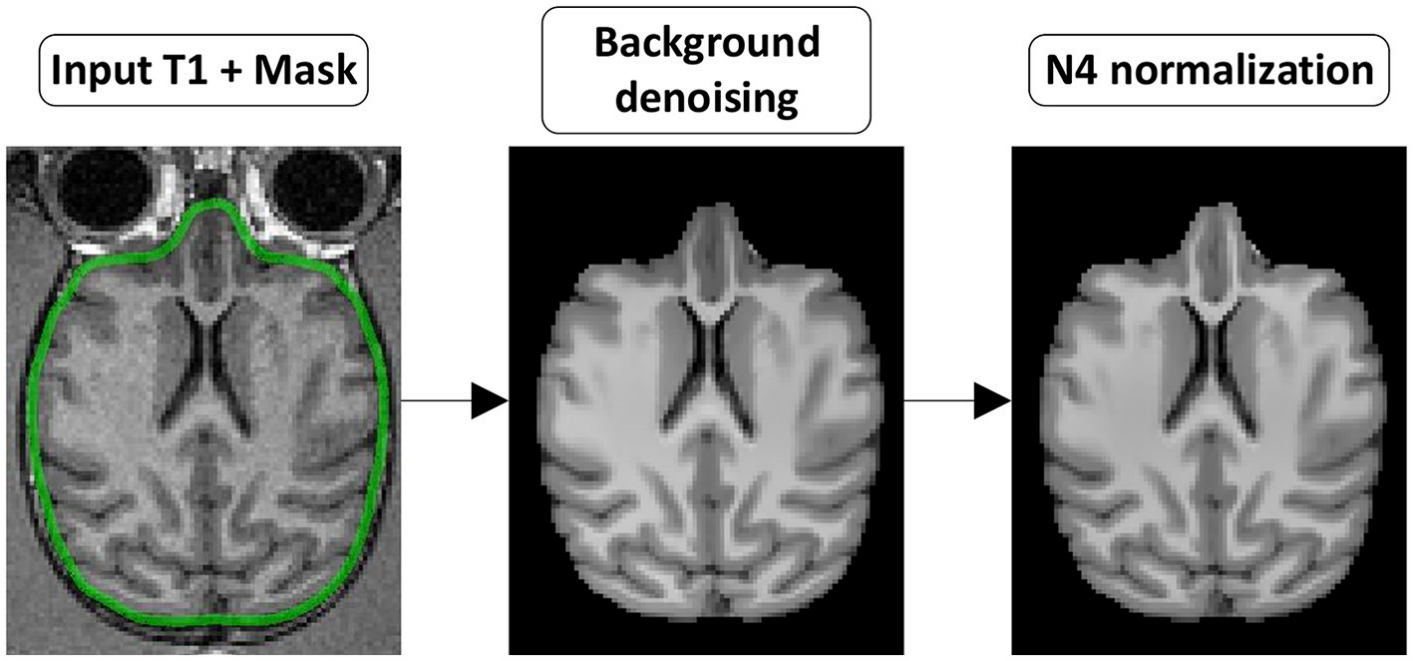
T1w preprocessing steps. (Background denoising) The T1w image is denoised to remove background noise. (N4 normalization) The denoised T1w image is then corrected for intensity non-uniformity.

**Figure 4:**
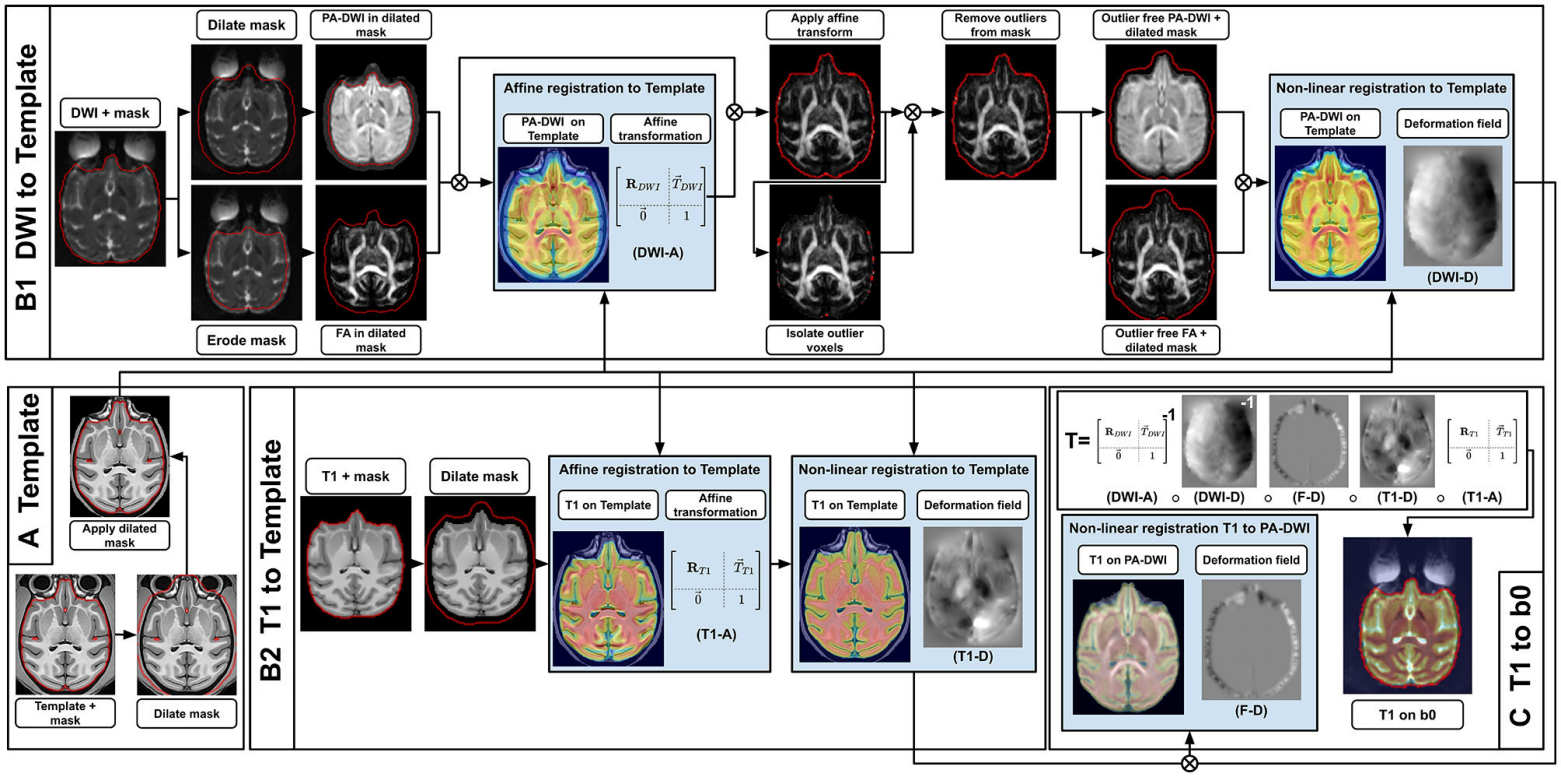
Registration sequence to align T1w images in the DWI space, using an intermediary high-resolution high-quality T1w template. First **(A)**, the template mask is dilated and applied to the template to hide most non-brain voxels while still keeping contour voxels between the brain and the skull, which improves alignment. Then, both the subject's T1w and DWI are registered to the template **(B1, B2)**. For the DWI **(B1)**, the brain mask in DWI space is dilated and the PA-DWI is computed inside it, while also eroded to compute the DTI-FA. The latter is done to exclude noisy voxels located around the brain volume which badly influence the registration. The PA-DWI and FA are used as a proxy to affinely register the DWI to the template (DWI-A). Next, the affine transformation (DWI-A) is applied to the DWI. Outlier voxels identified based on a DTI fit residuals and its agreement with the acquired b0 are then removed from the brain mask. This gives us the ability to compute an ”outlier free” PA-DWI and DTI-FA, which are used to non-linearly register the DWI to the template (DWI-D). For the T1w **(B2)**, the sequence of operations is simpler. After dilating the T1w mask, the T1w is registered affinely (T1-A) and non-linearly (T1-D) to the template. Finally **(C)**, from the template-registered DWI and T1w, a final non-linear transformation is computed to bring the T1w into the DWI space (F–D). The complete transformation from T1w space to DWI space (T) makes it possible to get masks and tissue maps required for further processing and analysis. Also, intermediary transformations between both modalities and the template (DWI-A + DWI-D and T1-A + T1-D) can be used to get averaged statistics in the subject space.

**Figure 5:**
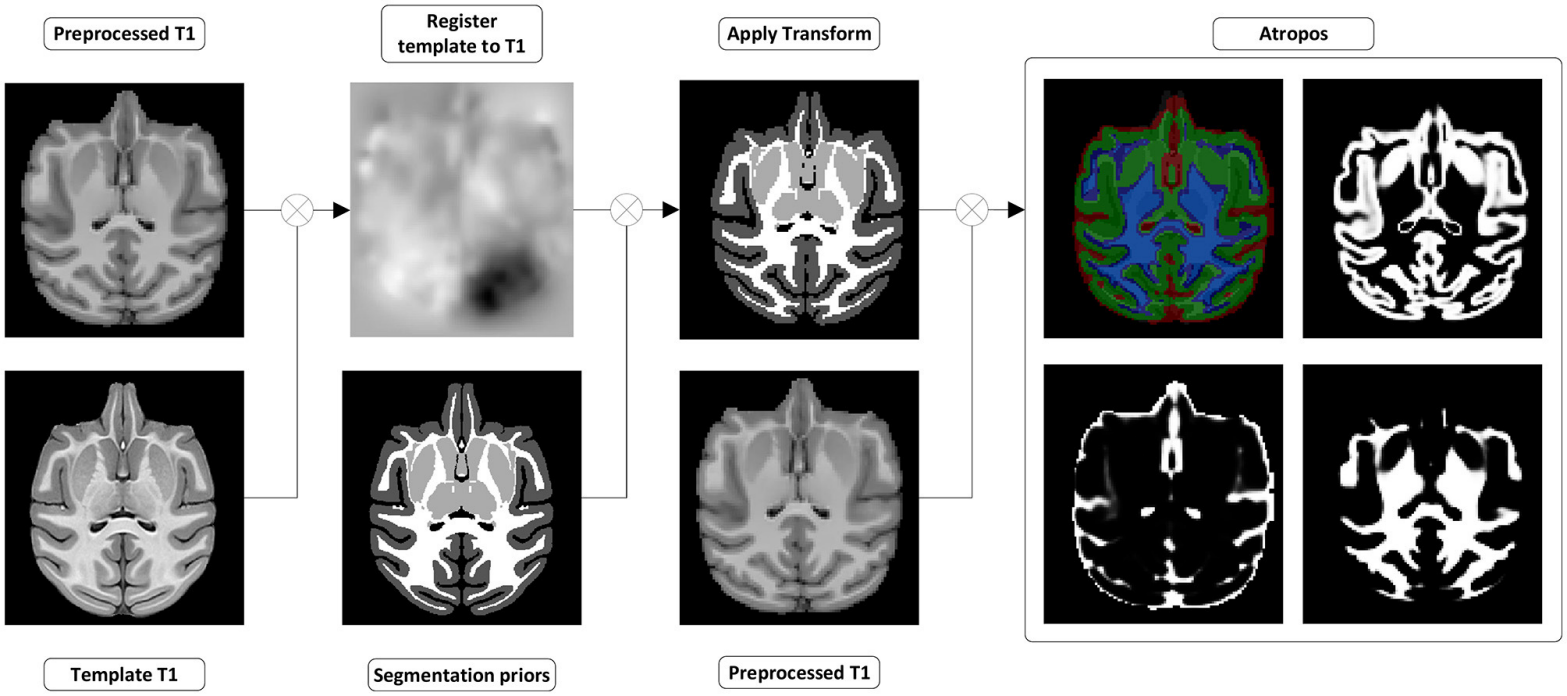
Tissues and white matter segmentation steps. (Register template to T1w) A transformation from the template to the preprocessed T1w image is computed. (Apply transform) The transformation is used to move the segmentation prior into the preprocessed T1w space. (Atropos) ANTs *Atropos* is run with the transformed segmentation priors, to compute segmentation masks and partial volume fraction maps of WM, GM, and CSF from the preprocessed T1w.

The following section presents all steps currently implemented in *versaFlow*, which are also available in our processes and workflows library. Even though we highly recommend using them all in the order prescribed to process raw anatomical and diffusion-weighted images, their execution is by default optional and can be individually turned on and off using the Nextflow configuration file, for instance, to allow the computation of the models and measures on already preprocessed data or to shorten the execution time by turning off steps deemed unnecessary after input data quality control. Visual descriptions of the processing workflows included in versaFlow can be found in Figure 2 for DW images preprocessing, Figure 3 for T1w images preprocessing, Figure 4 for registration between MRI modalities and Figure 5 for tissue segmentation.

##### 2.1.3.1. Preprocessing

###### 2.1.3.1.1. Diffusion processes

**Background noise:** DW images are affected by background noise following either a rician (Gudbjartsson and Patz, 1995) or a chi-squared distribution (Luisier et al., 2012) and more often than not present quite poor signal-to-noise ratio. Some patch-based algorithms (Manjón et al., 2008) enable the suppression of such types of noise but require long execution times and vast amounts of memory space, which can be cumbersome given the large size of diffusion volumes. A good trade-off is to use a *Principal Component Analysis* (PCA) to extract new dimensions isolating the different sources of noise from the data. This technique has proven its capability of enhancing the quality of diffusion data, even if it does not take into account the true nature of the noise and only corrects for normally distributed distortions. Thus, we run *dwidenoise* (Veraart et al., 2016a,b; Cordero-Grande et al., 2019), a patch-based *Marchenko-Pastur PCA* (MP-PCA) denoising algorithm available in Mrtrix3 (Tournier et al., 2019), at the beginning of the preprocessing tree for diffusion, on the forward and reverse phase-acquired diffusion volumes, and *Non-Local Means* (Manjón et al., 2008) denoising on the reverse phase acquired b0 volumes. Novel techniques using self-supervised learning such as *Patch2Self* (Fadnavis et al., 2020) and deep convolutional neural networks (Kawamura et al., 2021) could also be easily integrated into our modular framework, but have been discarded from the current implementation since their effects have not been entirely quantified yet.

**b0 to b0 normalization:** Acquisition of high spatial and angular resolution diffusion images often results in increased duty-cycle, especially when performing diffusion sensitization at high b-values. This can cause heating of the *Radio-Frequency* (RF) transmit coils and gradient arrays, which translates into a drift of signal intensities over time (Vos et al., 2017). To compensate for this effect, each pair of forward and reverse acquired images are normalized to the mean value of the first group of b0 volumes found in the forward image. To better correct the diffusion-weighted volumes, a linear combination of the means of the groups of b0 volumes found before and after each of them is used. This behavior can be modified to accommodate datasets with different deviations between b0 volumes and diffusion images, such as taking only the b0 before or after a group of diffusion directions to compensate for the signal drift.

**Gibbs ringing:** Since the spatial MRI images are reconstructed by inverse Fourier transform from a subset of points sampled through k-space, images can suffer from ringing artifacts at the interface between different tissues (Czervionke et al., 1988). When uncorrected, this effect biases diffusion models and metrics. We thus include Gibbs ringing correction as a step just after background denoising, using *mrdegibbs* (Kellner et al., 2016) from Mrtrix3 (Tournier et al., 2019).

**Susceptibility:** Discrepancies in magnetic susceptibility between brain tissues and neighboring tissues and cavities—such as the sinuses and the eyes—induce large gradients in the local magnetic field, which causes distortions in the *B*_0_ field that spatially displaces the acquired signal. In NHP studies, those artifacts are particularly intense, due in part to the proportionally bigger sinuses and other fatty tissues located around the brain. To correct for those, we use *Topup* (Andersson et al., 2003) from the FSL (Smith et al., 2004) library. *Topup* is run on the extracted b0 of the forward and reverse phase acquisitions to correct for susceptibility-induced distortions. The extraction of b0 can be configured to one of the following options:

- Take the first b0 appearing in the DW images.
- Take all the b0 in the DW images.
- Take the averages of b0 in the DW images.
- Take averages of continuous series of b0 in the DW images.

Since *Topup* is not parallelized, the pipeline comes initially configured to extract the average b0 in both phase directions and hypothesizes the distortions to be static across the duration of the acquisition. For this study, this configuration has proven to provide a good approximation of the local magnetic field while substantially lowering the execution time of the algorithm. However, in the case of dynamic distortions where the local magnetic field changes between diffusion volumes, sampling more than one b0 per dataset is recommended.

Prior to running *Topup*, all volumes are conjointly registered, first separately for the forward and reverse acquisitions and then to an average template using a multivariate template approach (Avants et al., 2011a). To prevent instilling artificial distortions in the estimated susceptibility field, registration steps are limited to rigid transformations.

**Powder averaging DWI:** It is common in DWI to use a b0 volume, unweighted by diffusion and independent of the gradient's orientation, to perform tasks such as registration between modalities. While this technique works well on human brains, it has proven unstable in our study. We found a better target for this to be a *Powder Averaged DWI* (PA-DWI) (Kaden et al., 2016). Our implementation simply averages all gradient orientations given in the DWI volume to produce a single 3D image, which has proven sufficient for registration purposes, even on single-shell data with few directions spread across the sphere.

**Brain masking:** Most of the subsequent steps in the pipeline require a mask for better and/or faster computation, thus we compute a mask using FSL *bet* (Smith, 2002) on the mean b0 volume, using the *Topup* corrected b0 if it was run. However, the execution of *bet* on monkey images has proven unstable and often leads to inclusion in the computed mask of skull and background regions. To circumvent this problem, we highly recommend passing in as inputs either a mask aligned to the diffusion data or computed on the T1w anatomical image, that has been quality checked and manually fixed. In the latter case, the b0 brain mask will be used only to help register the T1w to the mean b0 image + PA-DWI (via ANTs; Avants et al., 2008 using a combination of rigid and affine transformations) to align the mask in the diffusion space.

**Motion and Eddy currents:** Eddy currents naturally occur in tissues at the MRI due to the rapid variations of the imaging gradients (Ahn and Cho, 1991). They cause local distortions in the magnetic field perceived by the proton spins, resulting in biased diffusion-weighted measurements. To correct for those, we use FSL *Eddy* (Andersson and Sotiropoulos, 2016; Andersson et al., 2016, 2017, 2018), which also corrects for subject motion. This step is parallelized both on the *Central Processing Unit* (CPU) (using openMP) and the *Graphical Processing Unit* (GPU) (using CUDA on an Nvidia graphics card). When using the latter, the execution is more efficient and one can also perform outlier detection and replacement, as well as slice-wise motion correction.

**Intensity non-uniformity:** MRI equipment—transmit (*B*^+^) and receive (*B*^−^) fields—is not perfectly accurate and images can suffer from variations in intensities that are not related to the diffusion process of the acquired tissues. Since the diffusion-weighted signal is typically normalized by the b0 through modeling, this effect is usually overlooked. However, in the presence of intense motion, when using a strong B_0_ field or if subsequent fitting does not consider a normalized signal, it can be beneficial to correct for it (Van de Moortele et al., 2005; Ugurbil, 2018; Moeller et al., 2021). To do so, we run *N4* (Tustison et al., 2010) intensity normalization from ANTs on the mean b0 of each diffusion volume to compute the intensity bias field and then apply it to each volume in the 4D DW image. Its usage is optional and turned off by default since the correction does affect the distribution of noise in the diffusion volumes and could lead to biases in further modeling (Tax et al., 2022).

###### 2.1.3.1.2. T1w processing

**Background noise:** Since the T1w image is composed of a single 3D volume, *Non-Local Means* denoising (Manjón et al., 2008) becomes tractable in a reasonable amount of time. It is run using a rician noise model and parallelized over multiple CPU cores, using Dipy's implementation (Garyfallidis et al., 2014). This step has proven to improve segmentation and registration (Constanzo et al., 2018; Theaud et al., 2020) and has thus been integrated as an optional step in the pipeline.

**Intensity non-uniformity:** As for diffusion, the T1w image is corrected for local intensity non-uniformity caused by the MRI equipment using ANTs *N4* (Tustison et al., 2010) intensity normalization algorithm.

##### 2.1.3.2. Upsampling

Once denoised, both the T1w image and the diffusion volumes are upsampled to a finer resolution using Dipy (Garyfallidis et al., 2014). This step enhances anatomical features present in the diffusion volumes, brings both T1w and diffusion on a common spatial grid—allowing to use masks and segmentations estimated on the T1w image to better condition diffusion models reconstruction and compute tissue-specific metrics—and is also known to improve the results obtained from tractography (Dyrby et al., 2014). It uses linear interpolation on DWI, T1w images, and *Partial Volume Fraction* (PVF) maps, nearest neighbor interpolation on masks, and multi-label interpolation on label maps. The pipeline's default configuration resamples all images to half the voxel size of diffusion volumes. The pipeline can also be configured to resample the data to the resolution of either the T1w or the DW image and to disable resampling altogether. For the latter, either the T1w volume or the maps computed upon it are resampled to DWI resolution for internal usage in subsequent pipeline steps.

##### 2.1.3.3. Registration of anatomical space to diffusion space

To bring tissue segmentation maps and anatomical priors into the diffusion space, the upsampled T1w image is registered to the upsampled DW image space using a template approach. First, both images are individually and precisely aligned to the *MNI Monkey Space* (Frey et al., 2011) T1w template (resampled to a resolution close to the T1w and the DW images) using ANTs and a sequence of rigid, affine, and non-linear transformations. By registering to an intermediary high SNR target, it is possible to minimize misalignment between tissues that tend to occur with direct approaches, at the cost of roughly doubling computation times.

Image similarity is assessed by the *Mutual Information* metric. Two proxy images are used to align the DW image to the template:

- The PA-DWI volume (Afzali et al., 2021). In comparison to the b0 volume (which is often used as proxy in human studies), given a sufficient number of directions, the PA-DWI image possesses more contrast between the WM and the GM, proving a better target for the alignment of the cortical band. By construction, at low angular resolution, its contrasts converge to that of the b0. Thus, using it for registration is hypothesized to give similar or better results than using the b0 image alone. Since this image contains the principal anatomical landmarks required for accurate registration over the whole brain, it is weighted at 70% of the similarity metric cost.
- The *Fractional anisotropy* (FA) from a DTI fit. This image has been proven to improve sub-cortical alignment (Sboto-Frankenstein et al., 2014) when performing registration to or from T1w space. Since its purpose is to align WM structure only, it is weighted at 30%. Moreover, voxels near the brain boundaries are excluded, where the FA map is under the noise floor and does not carry information useful for supporting the registration.

The transformation from anatomical space to diffusion space is composed following equation 1, where **T**_DWI_ is the transformation from diffusion space to the template, **T**_T1w_ is the transformation from anatomical space to the template, and **T**_mid_ is the final non-linear registration computed between the template registered T1w and DW images to align the fine subject specific details.


(1) $$T_{\text{T1w}\to \text{DWI}}=T_{\text{DWI}}^{-1}T_{\text{mid}}^{}T_{\text{T1w}}^{}$$

Once computed, it is applied to all segmentation masks using nearest neighbor interpolation, to label maps resulting from tissue and white matter segmentation using multi-label interpolation, and to other scalar maps using linear interpolation.

##### 2.1.3.4. Segmentation

**Tissue segmentation:** Segmentation is performed on the T1w image in the anatomical space using ANTs *Atropos* (Avants et al., 2011b). The default configuration of the pipeline uses *AFNI NMT V2.0* segmentation (Jung et al., 2021) priors aligned to the *MNI Monkey Space*, registered to each subject's T1w image by applying their respective transformation computed in the registration step. Tissue masks are extracted from the PVF maps generated by *Atropos* via thresholding. A safe white matter mask, exempt of partial volume effects with both CSF and gray matter, is computed for future usage such as limiting the number of possible voxels used to compute the single fiber response necessary for constrained spherical deconvolution.

**White matter parcellation:** The default white matter atlas available in the pipeline is the *UWDTI* atlas (Adluru et al., 2012), aligned to *MNI Monkey Space*. The transformation computed in the registration step is used to warp the atlas to subject space and is applied using MultiLabel interpolation.

##### 2.1.3.5. Reconstruction

The pipeline offers 3 different models for the reconstruction of the diffusion process. To enable its usage on low angular resolution data or data sampled across a few shells at low b-values, the single *Diffusion Tensor Imaging* (DTI) (LeBihan et al., 2001) model can be fitted. It requires a minimum of 6 directions, though a uniform sampling of gradients over the sphere is preferable. Signal contamination can be caused by non-gaussian processes occurring at high b-value from exchange and restriction, for example, resulting in a drop of SNR and a loss of precision in estimated diffusion properties (Basser and Jones, 2002; Jones and Basser, 2004; Jensen and Helpern, 2010; Jones, 2010b; Chung et al., 2013). To limit such contamination, only shells up to b = 1,300 s/mm^2^ are used. This can be modified by the user if need be, but such modification is not recommended when processing *in vivo* data acquired from healthy subjects. For data acquired on a greater number of shells, with higher b-values, such as *High Angular Resolution Diffusion Imaging* (HARDI) (Tuch et al., 2002; Hosey et al., 2005) and *CUbe and SPhere* (CUSP) (Scherrer and Warfield, 2012), two higher-order models are available: the *fiber Orientation Distribution Function* (fODF) through *Constrained Spherical Deconvolution* (Tournier et al., 2007) (CSD) and the *DIstribution of Anisotropic MicrOstructural eNvironments in Diffusion-compartment* (Scherrer et al., 2016) (DIAMOND), a multivariate gamma distribution over tensors. For recommendations on optimized acquisition sequences and parameters to use with those higher-order models, we refer the reader to the following publications (Scherrer and Warfield, 2012; Jones et al., 2013; Genc et al., 2020).

##### 2.1.3.6. Diffusion metrics

**Figure 6:**
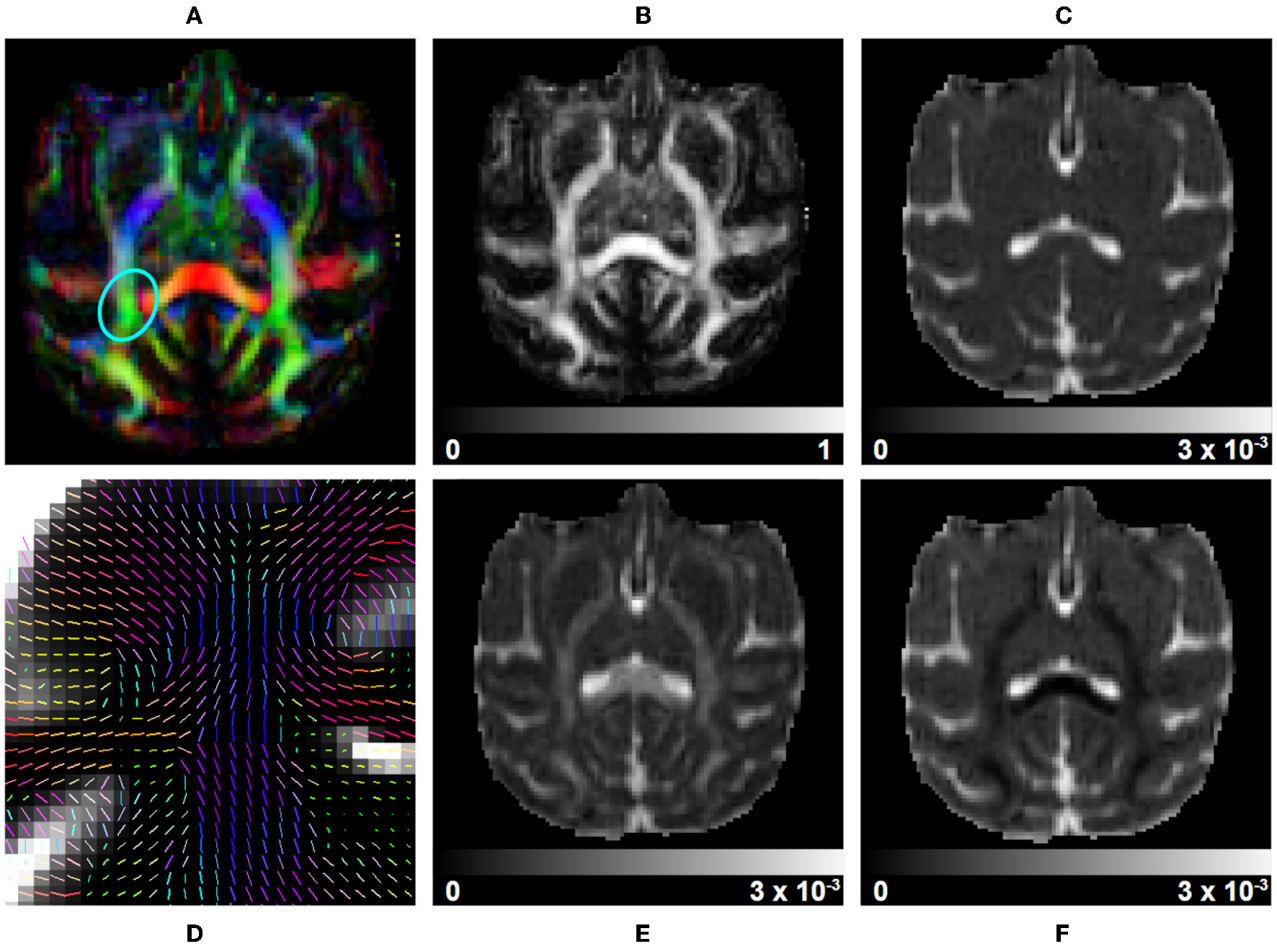
DTI measurements on a subject selected from the Aix-Marseille site. **(A)** RGB map, **(B)** fractional anisotropy (FA), **(C)** mean diffusivity (MD), **(F)** radial diffusivity (RD), **(E)** axial diffusivity (AD), and **(D)** the principal direction of the estimated tensor in the centrum semiovale [region circled in blue in **(A)**].

**DTI fit:** *Diffusion Tensors* (DT) are computed using Dipy and a *Weighted Least Square Fit*. In addition to the tensors, eigenvalues, and eigenvectors, it outputs *Axial Diffusivity* (AD, λ1) and *Radial Diffusivity* (RD, μ(λ_2_, λ_3_)), *Fractional Anisotropy* (FA) and *Geodesic Fractional Anisotropy* (GFA), and the mode and norm of diffusion tensors. Figure 6 presents part of these metrics. It also allows the output of validation maps, such as the residuals of the fit, the standard deviation across diffusion volumes displaying pulsation and misalignment artifacts, and a map of physically implausible voxels, representing the areas where the diffusion-weighted signal presents intensities higher than its associated average b0 signal.

**Figure 7:**
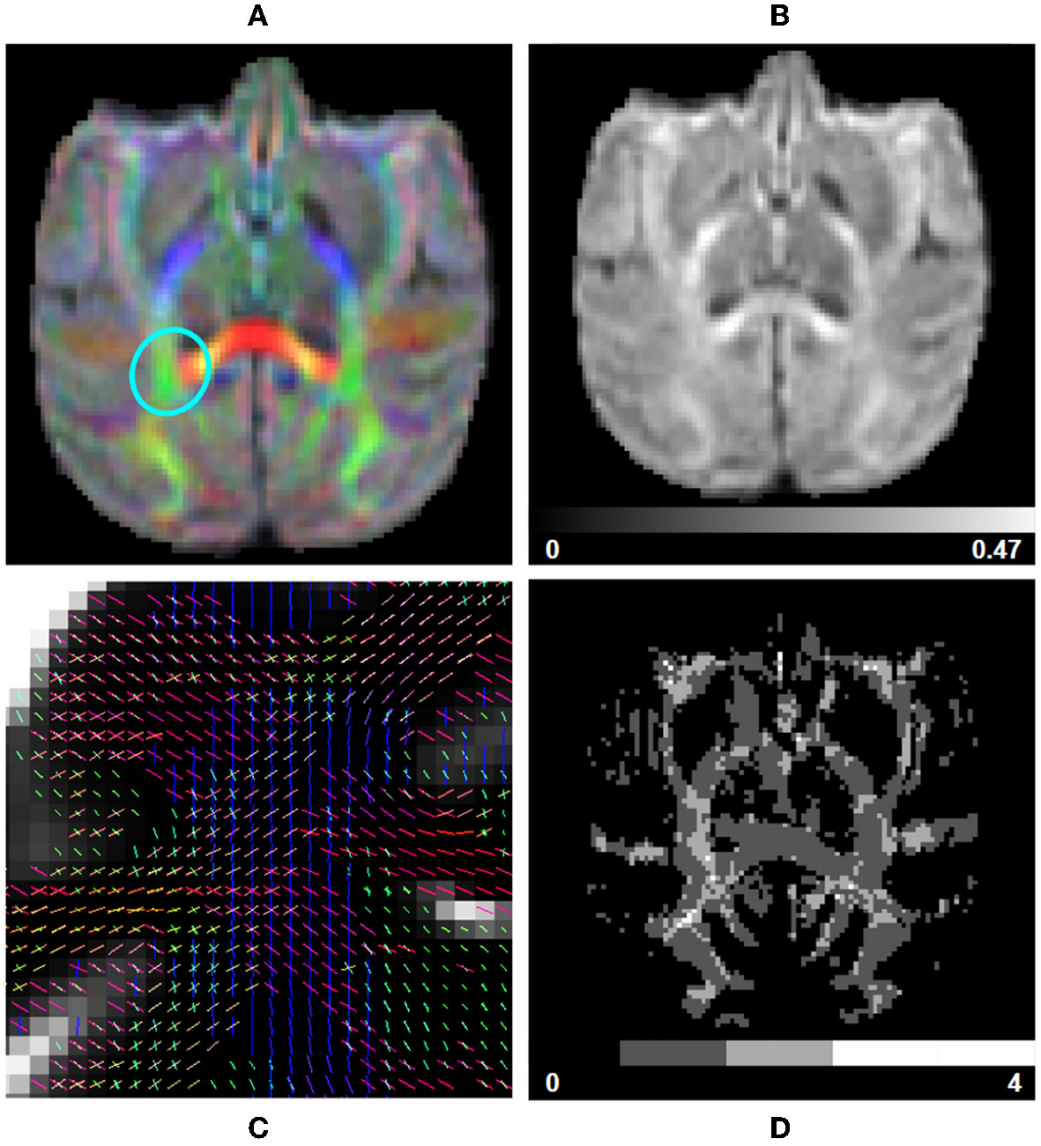
fODF measurements computed using data from a subject from the Aix-Marseille site. **(A)** RGB map, **(B)** total apparent fiber density (AFDt), **(D)** number of fiber orientations (NuFO), and **(C)** fODF peaks in the centrum semiovale [region circled in blue in **(A)**].

**CSD fit:** The response function and spherical deconvolution fit are also computed using Dipy. If a tissue segmentation is available at this step in the pipeline and the input DW image contains multiple b-value shells, a *Multi-Shell Multi-Tissue* (MSMT) approach is used (Jeurissen et al., 2014). Otherwise, the pipeline reverts to using a *Single-Shell Single-Tissue* (SSST) algorithm (Tournier et al., 2007). The WM response function is computed following an approach similar to the one used in human studies, with equivalent parametrization. It is estimated from a subset of single-fiber white matter voxels (200 per default) which are presenting a sufficiently high FA value (between 0.55 and 0.75 per default) and reside well inside the white matter mask computed previously. The peaks are then extracted from the fitted fODF, selecting only the ones presenting an amplitude higher than 1.5 times the maximal amplitude in isotropic voxels (Dell'Acqua et al., 2012) (Figure 7C). The latter are obtained using thresholds on the *Mean Diffusivity* (MD) (≥2.6*e*−3 mm^2^/s) and FA (≤ 0.15). Those thresholds are also used to compute the *Number of Fibers Orientations* (NuFO) and the *Apparent Fiber Density* (AFD) maps of voxel-based *Total* (AFDt, first SH coefficient), *Sum* (AFDs, integral on the sphere), and *Maximal Apparent Fiber Density* (AFDmax) (Dell'Acqua et al., 2012; Raffelt et al., 2012), as well as the RGB mapping of orientations on the sphere (Figures 7A, B, D).

**Figure 8:**
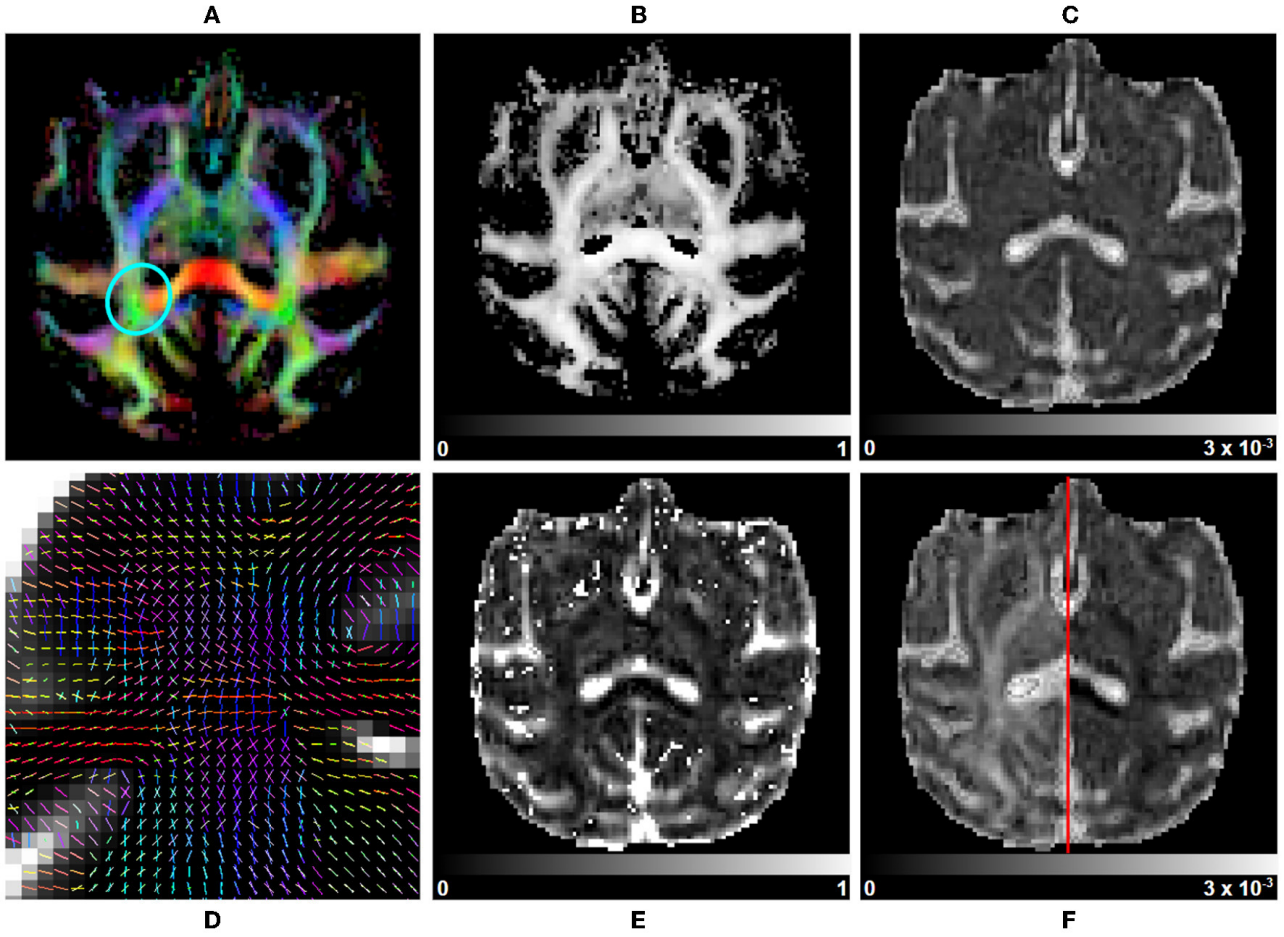
Multi-tensor measurements on a subject selected from the Aix-Marseille site. **(A)** RGB map, **(B)** maximum fascicle FA (max fFA), **(C)** mean diffusivity (MD), **(F)** axial diffusivity (AD) on the left, radial diffusivity (RD) on the right, **(E)** free water fraction (fFW), and **(D)** principal direction of the estimated mean tensor of each fascicle in the centrum semiovale [region circled in blue in **(A)**].

**Multivariate gamma distribution tensor fit:** The Multi-Tensor fit is acquired using DIAMOND (Scherrer et al., 2016), configured as described in further sections. Classical tensor measures (FA, AD, RD, MD, and RGB) are estimated on each fascicle separately and are used to compute their associated average, median, and maximum counterparts over fascicles. Maps of the fraction of each fascicle per voxel and of their main peaks are generated in addition. Other statistical quantities such as the mean and standard deviation of isotropic and anisotropic diffusivities (Reymbaut and Descoteaux, 2019) can also be computed. A subset of metrics is displayed in Figure 8.

### 2.2. Configuration automation

To make our pipeline as easy to use as possible, and to disentangle its input configuration from the data acquisition specification as much as possible, a great deal of care was given to automatize algorithm parametrization. Much information about the spatial positioning and the scale at which the imaging was performed can be inferred from the image itself, looking at its affine transformation property. Tools such as *dcm2niix* (Li et al., 2016) also allow the generation of a .json metadata file, containing even more imaging parameters extracted from the *DICOM* files, such as phase encoding direction, the order of slice acquisition, echo time, and repetition time, to name a few. Our implementation takes those parameters into account to optimally determine configurations at execution.

#### 2.2.1. Topup, Eddy, and ANTs

The *Topup* configuration we use is based on the *b0b20 macaque.cnf* configuration, from the NHP-HCP minimal processing pipeline (Autio et al., 2020). However, instead of defining the susceptibility field knot spacing and the blur window size in millimeters, we use non-dimensionalized ratios that are multiplied by the input image's voxel size. This allows the parameters to adapt themselves to the input spatial resolution. We also use metadata information to automatically determine the phase encoding direction and readout time of b0 images input into *Topup*.

As for *Topup*, we non-dimensionalize millimetric input parameters for *Eddy*, as well as for calls to various algorithms within the ANTs toolkit. For *Eddy*, we also use information from the metadata to specify the slice ordering of the DW volumes when using the outlier replacement option or the slice-to-volume registration corrections.

#### 2.2.2. Diffusion models

The complexity of diffusion models is determined at execution from the diffusion sampling strategy used when acquiring the DW images. For spherical harmonics models, the parameter to optimize is the reconstruction order. Its dependence on the number of independent gradient directions *N*_*i*_ is given by Equation 2 for a symmetric real harmonics basis (Descoteaux et al., 2006) and by Equation 3 for a full real harmonics basis (Bastiani et al., 2017).


(2) $$O_{\text{sym}}=\left\lfloor -3\;+\;0.5{\left(1\;+\;8N_{i}\right)}^{\frac{1}{2}}\right\rfloor$$

(3) $$O_{\text{full}}=\left\lfloor N_{i}^{\frac{1}{2}}-1\right\rfloor$$

For multi-shell samplings, the order is determined individually per shell and the minimum is selected to configure the algorithm. Compartment-based models such as DIAMOND are optimized on two fronts: the number of compartments to estimate and the complexity of the microstructural model. In our implementation, we consider the optimal starting model as:

- Three WM fascicles compartments at most in each voxel, to capture crossings.
- Non-constrained cylinder as the single fascicle model (6 diffusion tensor parameters + 1 fraction, per fascicle compartment).
- One free water compartment in each voxel, to reduce partial volume effects in estimated fascicle compartments (2 parameters).

This model requires at least 23 gradient directions to be correctly fitted. Given *N*_*f*_, the number of independent gradient directions in all shells of the input image, two cases are possible. First, *N*_*f*_ can be under 23 directions, in which case the complexity is reduced according to the following order of priority:

- Lowering the single fascicle model complexity.
- Lowering the number of fascicle compartments (up to 2).
- Removing the free water compartment.
- Lowering the number of fascicle compartments (up to 1).

Instead, if *N*_*f*_ is over 23 directions, the model's complexity is iteratively increased according to the following order of priority:

- Increasing the single fascicle model complexity.
- Adding restriction and hindrance estimation.
- Increasing the number of fascicle compartments.

This whole optimization process is optional, so users can force a particular configuration for their models and the pipeline will try to fit them even if the number of available directions is under the requirements. Note, however, that in this case, the uniqueness of the fitted model cannot be ensured; future usage of the reconstruction results must be done with care.

### 2.3. Study design and data selection

This study evaluates variability in diffusion-weighted measurements across sites available in the PRIME-DE project, as well as between human MRI scanner models and vendors used to acquire the images. From the 25 sites in the database, 8 gathered diffusion data, and only 4 have a fair number of subjects (*N* ≥ 4): Aix-Marseille University (Aix-Marseille, 4 subjects, Siemens Prisma scanner), University of California, Davis (UC-Davis, 19 subjects, Siemens Skyra scanner), Mount Sinai School of Medicine—Philips (Sinai-Philips, 9 subjects, Philips Achieva scanner), and Siemens (Sinai-Siemens, 6 subjects, Siemens Skyra scanner). All subjects are Macaca Mulatta primates, anesthetized before acquisition. The scan sequence parameters can be found in Table 1. All datasets were acquired using 3T systems, with DWI at spatial resolutions from 0.7 to 1.4 mm^3^–isotropic or rectangular voxels—and anatomical images from 0.3 to 0.8 mm^3^ isotropic voxels.

**Table 1:** Acquisition parameters for the sites of the PRIME-DE database containing diffusion data.

**Site**	**Scanner**	**# subjects**	**T1w**	**DWI**			
			**Voxel size**	**Voxel size**	**b-values**	**# directions**	**# b0**
Aix-marseille	Siemens Prisma 3T	1	0.8 mm	1.25 mm	250	6	5
			0.8 mm	1.25 mm	1,000	64	
			0.8 mm	1.25 mm			
		1	0.8 mm	1 mm	250	6	5
			0.8 mm	1 mm	1,000	64	
			0.8 mm	1 mm			
		1	0.8 mm	1.25 mm	300	6	7
			0.8 mm	1.25 mm	1,000	32	
			0.8 mm	1.25 mm	2,000	64	
		1	0.8 mm	1 mm	500	6	5
			0.8 mm	1 mm	1,000	30	
			0.8 mm	1 mm	2,000	30	
UC-Davis	Siemens Skyra 3T	19	0.3 mm^a^	0.7 mm	1,600	60	6
			0.3 mm^a^	0.7 mm			
			0.3 mm^a^	1.4 mm			
Sinai-Philips	Philips Achieva 3T	9	0.3 mm	1 mm	1,000	120	2
			0.3 mm	1 mm			
			0.3 mm	1 mm			
Sinai-Siemens	Siemens Skyra 3T	6	0.5 mm	1 mm	1,000	80	10
			0.5 mm	1 mm			
			0.5 mm	1 mm			

The subject from Aix-Marseille in the red box was used to create snapshots presented in Figures 6–8.

^a^T1w anatomical images were acquired at 0.6 mm isotropic with zero padding, for a final spatial resolution of 0.3mm isotropic.

### 2.4. Quality control

All input data including both DW and anatomical images went through quality control. Images were controlled for a variety of possible artifacts, including motion, ghosting, aliasing, Gibbs ringing, B_0_ and B_1_ field inhomogeneities, and magnetic susceptibility. After evaluation, images from Sinai-Siemens were excluded due to non-uniformity of contrasts across both DW and anatomical images from hyper-intensities caused by restraints positioned around the subject's head. The images from the 3 other sites had sufficient quality and were judged good for the study.

### 2.5. Brain masking

Before processing, brain masks were computed using the anatomical T1w images and *DeepBet v1.0* (Wang et al., 2021), a U-Net trained on macaque data from the PRIME-DE. All masks were quality controlled and manually fixed to prevent the exclusion of brain voxels and the inclusion of skull or eye voxels. The inclusion of this technology in the pipeline was considered but was ultimately rejected due to the required memory of its dependencies, practically doubling the size of the container required to run the pipeline.

Moreover, its usage was not proven to generalize to datasets other than the ones contained in the PRIME-DE, upon which it was trained, which could impede the pipeline's effectiveness. Also, its insertion near the beginning of the processing chain was deemed too risky, since its failure at providing a good brain mask would interfere with most of the subsequent steps.

### 2.6. Processing hardware

The processing was done separately for all 3 selected databases to evaluate the effectiveness of execution. We used 3 computing nodes of the Compute Canada Beluga cluster, each equipped with 2 Intel Gold 6148 Skylake CPU at 2.4 GHz clock speed, 186 Gb of *Random Access Memory* (RAM), and 4 NVidia V100SXM2 graphics cards with 16 Gb *Video RAM* (VRAM).

### 2.7. Pipeline configuration

The data from all 3 sites was processed using a common configuration. All sites acquired reverse phase encoded DWI volumes (same gradient sampling as the forward phase acquired volume), thus we ran *Eddy* using the whole reverse set of gradient directions and *Least-Square Resampling* (LSR) that preserves edges better in the resulting DWI. Images were resampled to a common isotropic spatial voxel size of 0.7 *mm*^3^. Most of the DW data available in the PRIME-DE was acquired on a single shell, thus CSD was run using an SSST approach. Similarly, DIAMOND was configured to estimate atmost 2 tensor populations per voxel, each parametrized by a *non-central gamma distribution over constrained cylinders* (second-order tensor with eigenvalues λ_1_ = λ_||_ and λ_2_ = λ_3_ = λ_⊥_). Minor modifications were made to Topup's configuration for UC-Davis, due to the magnitude of susceptibility-induced distortions: 3 intermediary resolutions were added to the subsampling pyramid, and the number of iterations at each level was increased. The configuration file (“aka: b0b20_versa_primede_uc_davis.cnf”) is available in Supplementary material.

### 2.8. Pipeline reproducibility

To evaluate correctly the potential variations in metrics between the several subjects of the PRIME-DE database, it is required that the processing carried on the data be as reproducible as possible. To this end, all algorithms that require the execution of a random process have seen their seed enforced to the same number across all subjects and sites. To evaluate the reproducibility, we did a test-retest analysis using the 4 subjects from the Aix-Marseille database, on which the pipeline was run 3 separate times using the same configuration and Singularity image. The *Image Intraclass Correlation Coefficient* (I2C2) (Shou et al., 2013) was computed using Equation 4 on relevant DTI, fODF, and multi-tensor metrics.


(4) $$\begin{array}{l} \text{I2C2}=1-\frac{\sum_{s\in S}N_{s}-1}{\sum_{s\in S}\left(N_{s}-1\right)}\underset{s\in S}{\sum^{\text{​}}}\underset{ss\in s}{\sum^{\text{​}}}\underset{v\in I_{ss}}{\sum^{\text{​}}}\frac{{\left(I_{ss}(v)-{Î}_{s}(v)\right)}^{2}}{{\left(I_{ss}(v)-Î(v)\right)}^{2}} \end{array}$$

Here, *I*_*ss*_ refers to a modality, mask, or measurement of a specific subject *s* and session *ss* to evaluate, Î_s_ is the mean image for a specific subject *s* taken over all its sessions, and Î is the mean image taken over all sessions and all subjects.

### 2.9. Data quality evaluation

To quantify the increase in quality of the processed data, two measures, *Signal-to-Noise Ratio* (SNR) and *Contrast-to-Noise Ratio* (CNR), were computed inside each region of the registered tissue maps on the mean b0 volume of the diffusion-weighted images. *Topup* correction and upsampling were performed on the raw data, to allow for the use of the tissue maps when computing their SNR and CNR. Thus, our method does not quantify the effect of those steps on the amelioration of the data quality.

To calculate SNR, two different approaches were used to quantify the noise standard deviation using the b0 data. For DW images containing multiple b0 volumes, intermediate noise maps were extracted by subtracting each possible permutation of two b0 volumes. Those maps were then averaged to obtain a mean noise map, which was used to compute the noise standard deviation in each region. SNR was then calculated using equation 5 (Narasimhan and Jacobs, 2002). For DW images with a single b0 volume, a background mask was extracted using the technique presented in Balan et al. (2012) and the noise standard deviation was calculated from the b0 voxels that overlapped the mask. SNR was calculated using Equation 6. In both Equations 5 and 6, *b*0 refers to the b0 image, *N* to the extracted mean noise map, *T*_mask_ to the mask of the tissue of interest, and *B*_mask_ to the image background mask. μ_*M*_(*I*) and $\sigma_{M}^{2}$(*I*) respectively refer to the mean and variance of all voxels of an image *I* which overlapped a mask *M*.


(5) $$\begin{array}{l} SNR\left(b0,\;N,T_{mask}\right)=\frac{\mu_{T_{mask}}\left(b0\right)}{\sigma_{T_{mask}}^{2}\left(N\right)} \end{array}$$

(6) $$\begin{array}{l} SNR\left(b0,\;T_{mask},B_{mask}\right)=\frac{\mu_{T_{mask}}\left(b0\right)}{\sigma_{B_{mask}}^{2}\left(b0\right)} \end{array}$$

CNR was computed using the b0 by comparing white matter with gray matter regions using Equation 7.


(7) $$\begin{array}{l} CNR\left(I,\;WM_{mask},GM_{mask}\right)=\frac{\left|\mu_{WM_{mask}}\left(I\right)-\mu_{GM_{mask}}\left(I\right)\right|}{\sigma_{WM_{mask}}^{2}\left(I\right)+\sigma_{GM_{mask}}^{2}\left(I\right)} \end{array}$$

We also performed a variability study in tissue maps (WM and GM) of several metrics extracted from the diffusion models available in the pipeline. Using the average over sessions of subjects, we analyzed inter-subject variability separately for each database (Equation 8), intra-vendor variability for Siemens scanners (UC-Davis and Aix-Marseille) (Equation 11), and inter-vendor variability using all sites (Equation 10). Since UC-Davis and Sinai-Philips presented multiple time points per subject, intra-subject variability was also computed (Equation 9).


(8) $$\text{BS-CV}(\left\{{\hat{S}}_{1},\ldots ,{\hat{S}}_{n}\right\}\in D_{i},M)=\frac{\sigma_{M}(D_{i})}{\mu_{M}(D_{i})}$$

(9) $$\begin{array}{l} \text{IS-CV}\left(\left\{S_{j,1},\ldots ,S_{j,k}\right\}\in S_{j},M\right)=\frac{\sigma_{M}\left(S_{j}\right)}{\mu_{M}\left(S_{j}\right)} \end{array}$$

(10) $$\text{BV-CV }(\left\{{\hat{S}}_{D_{1},1},\ldots ,{\hat{S}}_{D_{1},n_{1}},\ldots ,{\hat{S}}_{D_{v},n_{v}}\right\}\in V,M)=\frac{\sigma_{M}(V)}{\mu_{M}(V)}$$

(11) $$\text{IV-CV }(\left\{{\hat{S}}_{D_{1},1},\ldots ,{\hat{S}}_{D_{1},n_{1}},\ldots ,{\hat{S}}_{D_{v},n_{v}}\right\}\in V_{i},M)=\frac{\sigma_{M}(V_{i})}{\mu_{M}(V_{i})}$$

Here, *S*_*j*_ denotes a set of all repetitions of an image (T1w, DWI, masks, and measures) for a single subject, *S*_*j, k*_ a repetition in that set, and Ŝ the set averaged over repetitions. *D*_*i*_ is a set of images associated with a single acquisition site (Aix-Marseille, Sinai-Philips, or UC-Davis), *V*_*i*_ is a set of images associated with a vendor (Philips, Siemens), and *V* is the set of all repetitions from all subjects across all vendors. *M* is a mask defining a region of interest in which the variation measure is evaluated.

## 3. Results

### 3.1. Processing time

**Figure 9:**
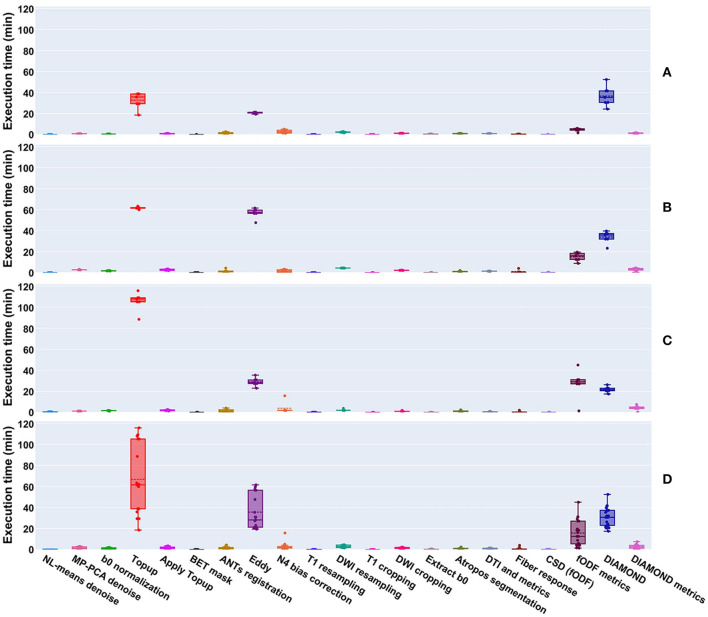
Execution time of processes run on datasets from the **(A)** Aix-Marseille, **(B)** Sinai-Philips, and **(C)** UC-Davis sites. **(D)** Presents the global duration per process over all sites. Most algorithms took less than a few minutes to execute, with the exception of *Topup* (single threaded), *Eddy* (parallelized on the GPU using CUDA 9.1), DIAMOND (parallelized on the CPU), and the computation of fODF metrics (single threaded).

Per-processing execution time is displayed in Figure 9 separately for each site. Most of the processing time was taken by 4 algorithms, namely *Topup* (~66 min avg), *Eddy* (~36 min avg), DIAMOND (~31 min avg), and fODF metrics computation (~15 min avg); those times are tightly related to the diffusion image resolution, here ranging from isotropic 1 mm^3^ to isotropic 1.25 mm^3^. Every other major step of the pipeline was executed in approximately 5 min or less. It took approximately 6 h to process the 4 datasets of the Aix-Marseille site, 25 h for the 27 (9 subjects, 3 sessions) of Sinai-Philips, and 34 h for the 38 (19 subjects, 2 sessions) of UC-Davis. Note that for UC-Davis, the number of iterations for *Topup* had to be increased, due to the intense susceptibility artifacts present in the images, thus drastically increasing the execution time.

### 3.2. Image quality

**Figure 10:**
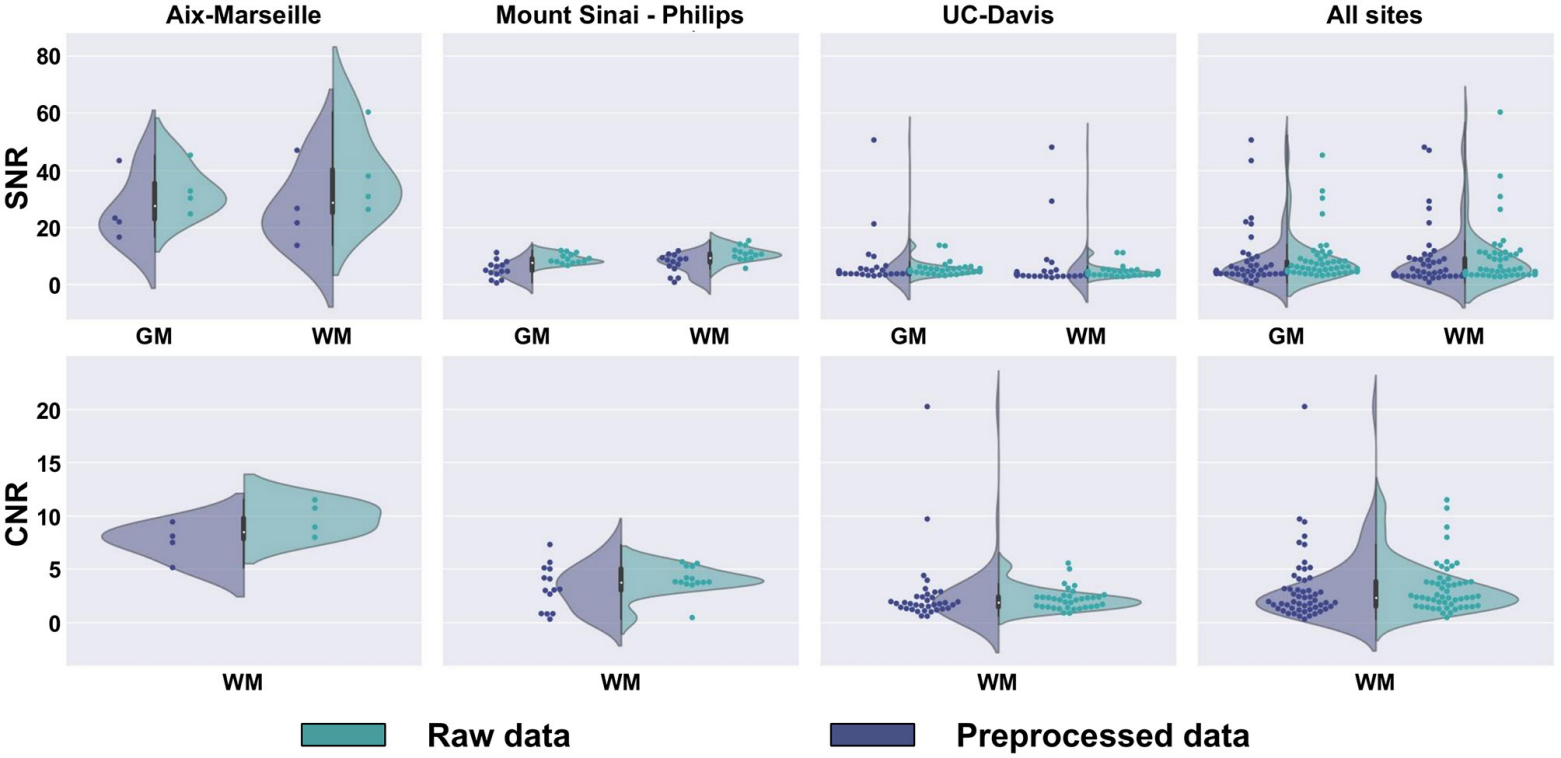
SNR and CNR distributions of b0 images. SNR is presented separately for (WM) white matter, (GM) gray matter, and (CSF) cerebrospinal fluid. CNR is only presented for the white matter, since the measure results from a comparison to gray matter image intensities.

SNR and CNR are presented in Figure 10, on the average b0 volume extracted from both the raw and preprocessed DW images. Except for UC-Davis, the SNR measure suggests an increase in image quality resulting from the different denoising algorithms used in the preprocessing part of the pipeline. For Sinai-Philips and UC-Davis, the SNR distribution displays less variance after preprocessing, with a substantial increase in mean SNR for Sinai-Philips. Similarly, distributions of CNR display less variance after preprocessing for Sinai-Philips and UC-Davis. Mean CNR is also increased for Aix-Marseille and Sinai-Philips.

### 3.3. Reproducibility

Table 2 shows reproducibility scores on relevant output images of the pipeline. For classical diffusion models (DTI and fODF), as well as for the anatomical mask computing and registration processes, reproducibility is high, with I2C2 scores of 98% or higher. Scores for the DIAMOND model averaged around 97% for metrics computed over the fascicles (MD, AD, RD, FA, max fFA, and free-water fraction) and 91% for model selection, indicating that the outcomes of this algorithm are less reproducible. Gamma distribution parameters (kappa, kappaAD, hei, and heiAD) did not perform as well, with kappa and hei scoring 83% and heiAD and kappaAD scoring 52 and 63%.

**Table 2:** Reproducibility measures computed on images generated by the pipeline.

**Modality**		**Measure**	**I2C2**
Anatomy	T1w	WM PVF	0.997
	T1w	GM PVF	0.995
	T1w	CSF PVF	0.990
	T1w	Registration	1.000
Diffusion	DTI	MD	0.991
	DTI	AD	0.990
	DTI	RD	0.991
	DTI	FA	0.999
	CSD (fODF)	AFD	0.981
	CSD (fODF)	AFD total	0.997
	CSD (fODF)	NuFO	0.986
	DIAMOND	MD	0.962
	DIAMOND	AD	0.975
	DIAMOND	RD	0.956
	DIAMOND	FA	0.990
	DIAMOND	Max fFA	0.979
	DIAMOND	hei	0.831
	DIAMOND	heiAD	0.523
	DIAMOND	kappa	0.831
	DIAMOND	kappaAD	0.630
	DIAMOND	Fascicle fractions	0.968
	DIAMOND	Free-water fraction	0.981
	DIAMOND	Model selection	0.903

### 3.4. Variation of measurements

**Figure 11:**
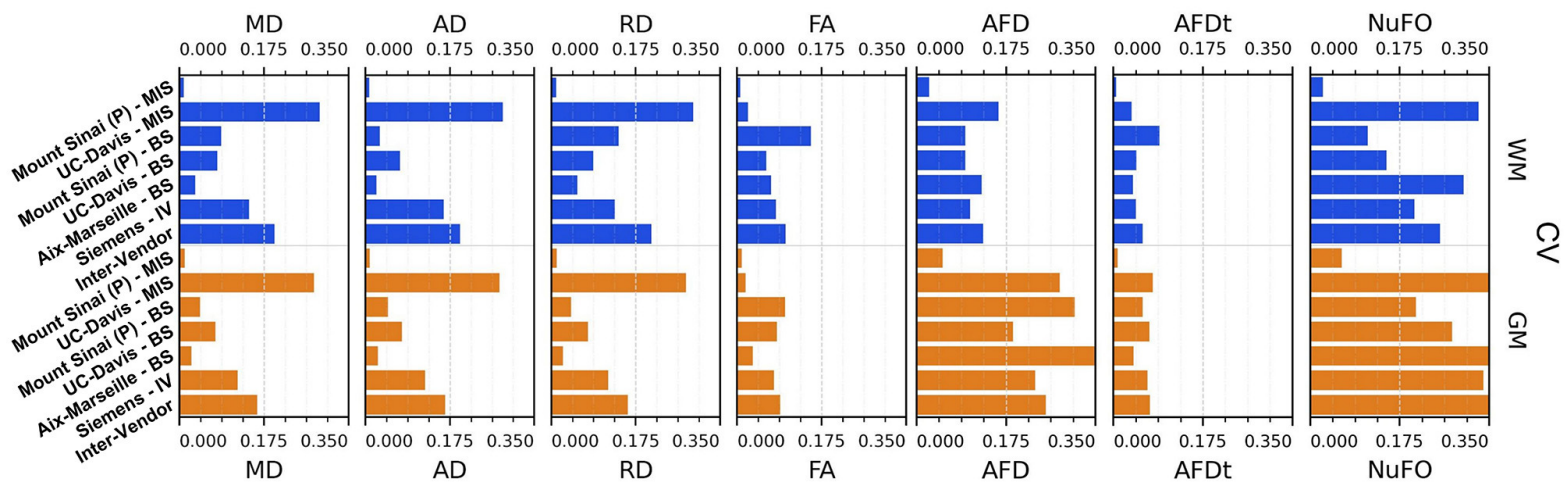
Coefficient of variation (CV)—mean intra-subject (MIS), inter-subject (BS), intra-vendor (IV), and inter-vendor (BV)—for DTI and fODF measurements: MD, mean diffusivity; AD, axial diffusivity; RD, radial diffusivity; FA, fractional anisotropy; AFD, max apparent fiber density; AFDt, total apparent fiber density; NuFO, the number of fiber orientations.

**Figure 12:**
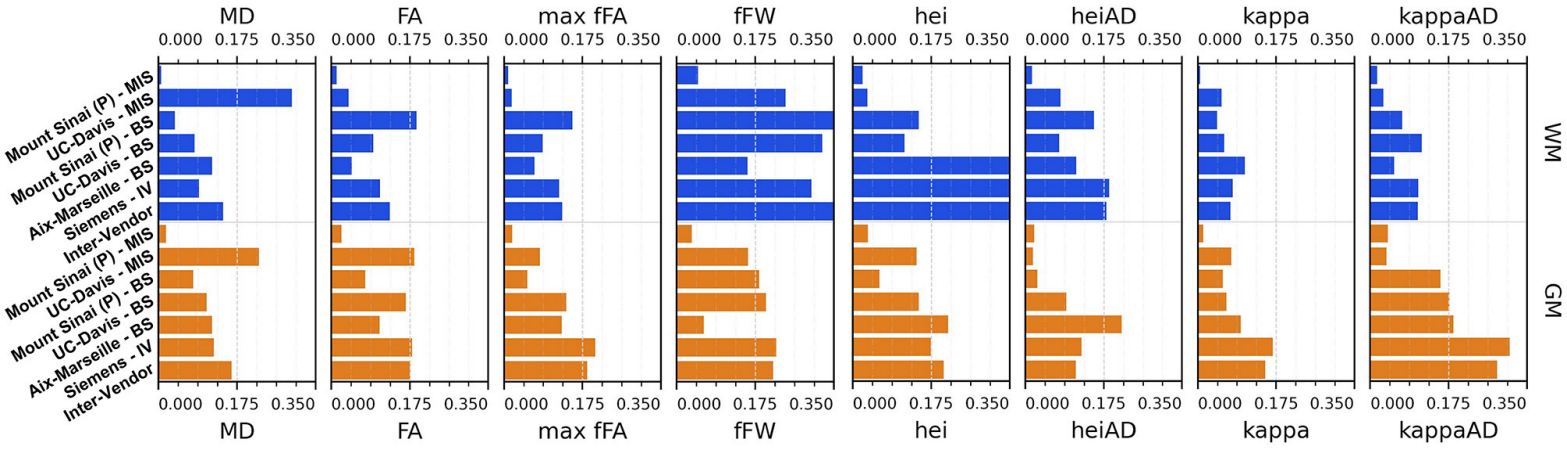
Coefficient of variation (CV)—mean intra-subject (MIS), inter-subject (BS), intra-vendor (IV), and inter-vendor (BV)—for diamond measurements and gamma distribution parameters: MD, mean diffusivity among fascicles; FA, mean fractional anisotropy among fascicles; max fFa, maximum fascicle FA; fFW, fraction of free-water; hei, heterogeneity index; heiAD, axial heterogeneity index; kappa, kappa parameter of tensor distribution; kappaAD, axial kappa parameter of tensor distribution.

Figures 11, 12 display the coefficients of variation—*Mean Intra-Subject* (MIS), *Inter-Subject* (BS), *Intra-Vendor* (IV), and *Inter-Vendor* (BV)—of several metrics computed by the pipeline; Figure 12 also displays the coefficients for the gamma distribution parameters estimated by the DIAMOND model, calculated in both white matter (WM) and gray matter (GM) masks.

The Sinai-Philips site presents the least mean intra-subject variability overall at ≤ 2%, except for NuFO, AFD, and *free-water fraction* (fFW), but the worst inter-subject variability in almost all metrics and parameters evaluated.

For DTI metrics, Aix-Marseille displays lower inter-subject variability in both white matter (≤ 4% in MD and RD and ≤ 7% in RD and FA) and gray matter (~3%) than Sinai-Philips. The portrait is reversed for fODF measurements, with the exception of AFDt where the variability observed is greater (more than twice for NuFO in WM). For metrics computed on the DIAMOND model, the variability is lower, with the exception of MD in WM and of all measures excluding fFW in GM. The variability is also higher for gamma distribution parameters, except for kappaAD and heiAD in WM.

**Figure 13:**
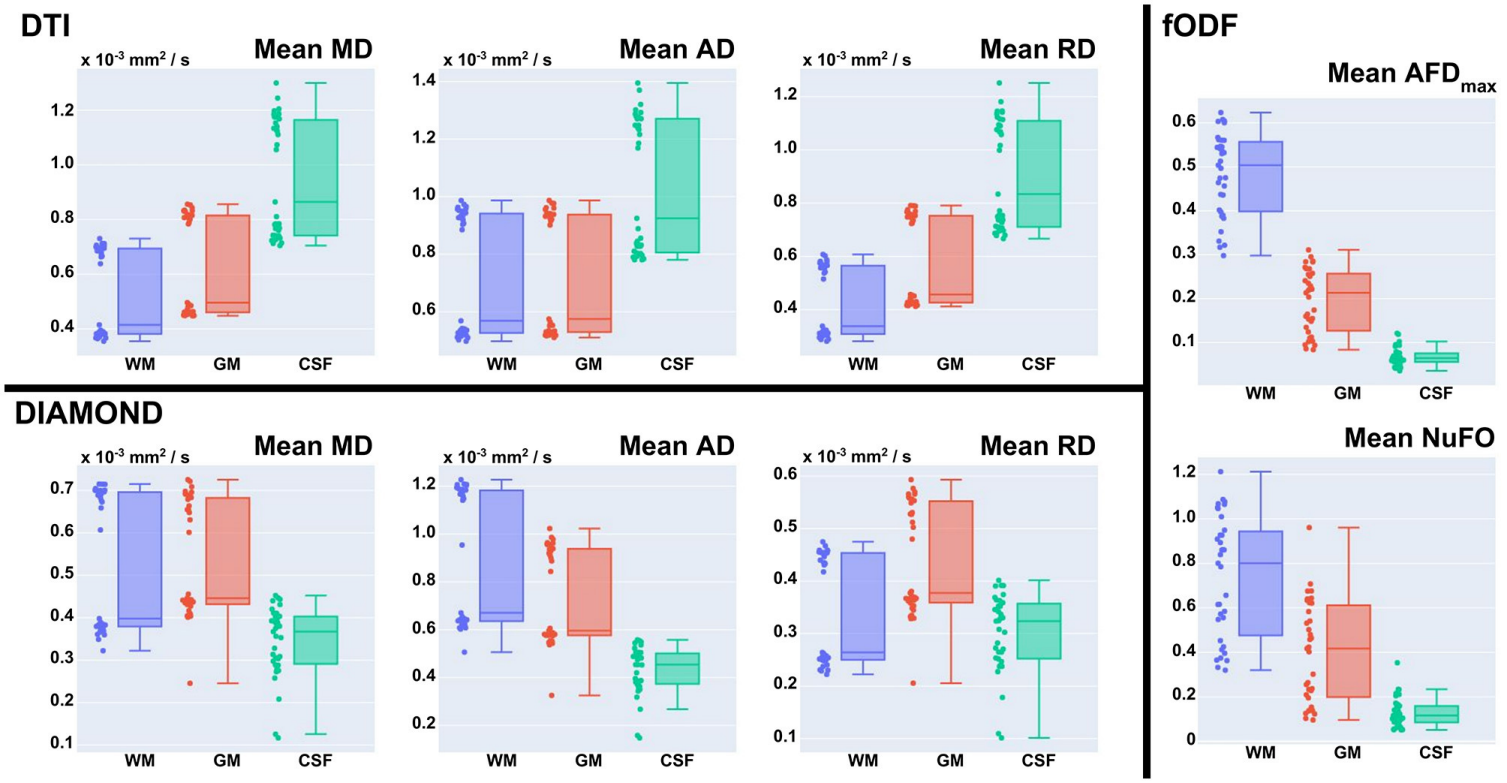
Clustering behavior of the acquisition sessions of the subjects from the UC-Davis site.

UC-Davis exhibits low inter-subject variability in DTI measurements at < 4% for MD, AD, and RD and approximately 6% in WM and 8% in GM for FA. In WM, fODF metrics such as AFDt and AFD have a similar behavior, with CV at 4 and 9%, respectively, while NuFO presents as the least reliable at 14%. Oddly, mean intra-subject variability is greater than inter-subject variability for this site for almost all quantities observed, except for fractional anisotropy (with the exception of the mean FA on DIAMOND fascicles), AFDt (in WM only) and free-water measurements, as well as for gamma distribution parameters excluding heiAD in WM and kappa in GM. With further inspections, we noticed that for all measurements exhibiting this trend, two clusters of similar measurements could be formed, corresponding each to one of the sessions at which the subjects' images were acquired, as presented in Figure 13.

Intra-vendor variability of Siemens scanners is computed on subjects from both Aix-Marseille and UC-Davis. For DTI metrics, variability in WM ranges from 12 to 14% for diffusivity measures and at around 8% for FA and is slightly lower in GM. For fODF, NuFO displays the highest variability (21% in WM and 32% in GM), followed by AFD (10% in WM and 22% in GM). Variability for AFDt is substantially lower at 4% in WM and 7% in GM. For measurements on the DIAMOND model in WM, MD is the least variable at 6% and fractional anisotropy scores 10%, 2% below the fascicle-based measure (max fFA). Results show that performance from those measurements is approximately two times more variable in GM. Free-water fraction is the most variable measure estimated at 28% in WM and 21% in GM. For gamma distribution parameters, the values ranged widely depending on the parameter and the tissue. WM kappa and kappaAD are the less variable at 7 and 10%, and hei and heiAD score 33 and 18%, respectively. For GM, the parameter with the highest variability is kappaAD at 32%, followed by kappa and hei at around 17% and heiAD at 13%.

Inter-vendor variability was consistently higher than intra-vendor on all DTI measurements, with an increase of 2% in the variability of FA and from 4 to 10% in diffusivity measures in both WM and GM. The same trend is observed for fODF metrics, AFDt displaying the less increase (2%) and NuFO the most (5%) in WM. For measures on the DIAMOND model, MD showed the highest increase (6% in WM and 3% in GM). FA and maxfFA variability remained stable, with a slight increase of 2% for the former and ~1% for the latter. Free-water fraction variability increase was substantial—approximately 5%. For gamma distribution parameters, a slight decrease of variability was observed for all parameters, except for hei which remained stable in WM and increased in about 3% in GM.

## 4. Discussion

### 4.1. Sources of variability in DWI

The greatest confounding factors in current DWI studies are acquisition harmonization and processing reproducibility. Acquisition harmonization refers to the capacity of data collection equipment and facilities to provide standard measurements independent of location, time, climate, equipment vendor, and model. These measurements need to be stable given a specific tissue or effect to probe. We know, from a plethora of multi-site, multi-vendor studies (Grech-Sollars et al., 2015; Fortin et al., 2017; Mirzaalian et al., 2017; Duchesne et al., 2019; Prohl et al., 2019; Tax et al., 2019), that using non-harmonized data can increase the variability of the measurements acquired at the scanner and potentially interfere with the statistical significance of analyses. One of the popular datasets, the *SIMON dataset* (Duchesne et al., 2019), has even shown that images from the same subject acquired at separate sites may lead to drastically different interpretations, even if the equipment manufacturer and MRI model are the same (in the case of differences in models and vendors, the results are even worse).

We see this effect clearly in our variability analysis of the data from the 3 sites of the PRIME-DE. To better identify its sources, we evaluated the coefficient of variability on 4 levels: mean intra-subject (MIS) on the Sinai-Philips and UC-Davis sites, inter-subject (BS) on all 3 sites, and intra-vendor (IV) on sites using Siemens scanners (Aix-Marseille and UC-Davis) and inter-vendor (BV) on all 3 sites. Looking at MIS-CV measurements for the Mount-Sinai (P) site only, we can expect that the effects of a subjects intrinsic variability and variability due to the environment should be weak—with CV reported in the range of a few percent for most metrics. This is in line with results previously reported in human studies (Grech-Sollars et al., 2015; Prohl et al., 2019). Variations between subjects (BS-CV) are significantly higher and should potentially be detected by the analyses using this cohort. However, those variations are masked by effects caused by differences in vendors (BV-CV) or in equipment provided by the same vendor (IV-CV). In the majority of metrics we studied, we can see 2 to 6 fold increases in variability when pooling data across scanners and vendors, suggesting that little to no sensitivity to differences between subjects should be expected if harmonization is not carried out. This is a typical trend in DWI and all multi-site and/or multi-vendor databases suffer from such variability to some extent (Grech-Sollars et al., 2015; Fortin et al., 2017).

Lowering intrinsic, environmental, site, and vendor variability can be done in a few ways. At the MRI, the adoption of standard acquisition sequences and the standardization of MRI equipment can be effective solutions, though the task to tackle is monumental. On one hand, MRI vendors are reluctant to standardize because novel proprietary techniques give them a commercial advantage. On the other hand, it has been difficult to gather consensus on acquisition protocols from the community—though international consortiums are converging to guidelines and good practices (Jelescu et al., 2023; Schilling et al., 2023)—partly because specific sequences serve particular research questions better, but also because the lack of gold standards in DWI makes it difficult to compare sets of good acquisition parameters. However, without some standardization, group efforts such as the PRIME-DE project—that pools data from multiple sites, often with reduced cohort sizes—cannot become fully relevant. As can be seen in Table 1, acquisition parameters vary widely from one site to another, especially when it comes to the sampling of diffusion gradients' directions. This has a direct impact on the validity of the reconstruction of diffusion models, since different b-values encode different processes in the imaged tissues (McKinnon et al., 2017; Veraart et al., 2019).

In our study, gradient sampling seems to have a limited impact on DTI measurements for Aix-Marseille, since shells with b-values higher than 1,300 s/mm^2^ are omitted. This, however, has a greater effect when comparing UC-Davis' data to data from other sites, since DTI data from UC-Davis was reconstructed from the only available shell at 1,600 s/mm^2^. This explains the 3 to 4 fold increase in Siemens intra-vendor and global inter-vendor variability relative to the average inter-subject variability of associated sites. The impact becomes greater when looking at fODF and multi-tensor reconstruction (DIAMOND), since those models make use of all b-values. This increases Aix-Marseille's MIS-CV since the analysis becomes a comparison between 2 subjects acquired with single-shell sampling and 2 others with multi-shell, the latter presenting a richer portrait of the diffusion process by the usage of higher gradient weighting. Both fODF and multi-tensor reconstruction are known to benefit from the higher b-values (Scherrer and Warfield, 2010, 2012; Taquet et al., 2015; Yang et al., 2015); the quantities estimated from those models are expected to vary in the case of a low b-value single-shell acquisition.

Another trend observed in our study indicating the need for guidelines and protocols is the high mean intra-subject variability observed for UC-Davis. While analyzing the data from this site, we noticed a strong discrepancy in diffusion measurements between the two sessions at which the images were acquired. This behavior could be the result of software or hardware upgrades, resulting in a suboptimal acquisition of the diffusion profile. The only quantities where this was not observed are fractional anisotropies (FA and max fFA) and total apparent fiber density (AFDt). Nevertheless, this makes the usage of separate sessions from UC-Davis impossible in a study of diffusion measurements as the variability would diminish any statistical power. We hope that in future, good quality control could be integrated into the scanners to prevent or correct the acquisition of images presenting this kind of behavior.

For now, DWI data harmonization at the scanner is not always possible, but it can be obtained to a certain degree with the use of software to normalize the statistical distribution of subjects in a given database. This usually requires hypotheses on how those distributions should behave given a set of metrics and try to compensate for the sources of site-dependent or scanner-dependent variability, while conserving each subject's intrinsic variability and the variability between them. Good solutions are available today, capable of reducing the effects induced by the acquisition software and technology (Vollmar et al., 2010; Grech-Sollars et al., 2015; Fortin et al., 2017; Tax et al., 2019), as well as signal deviation occurring from environmental differences (temperature, humidity, time of day, etc.) (Meyer et al., 2016; Book et al., 2021). However, a lot of work still needs to be done to improve their implementation and to properly quantify their effectiveness. Databases such as the PRIME-DE are prime candidates for this kind of studies since NHP data are acquired using standard human scanners and sequences, as well as similar head coils, but their environment and vitals can be more easily controlled and monitored. Repetition of measurements should present lower variability and thus environment, scanner, and vendor-dependent effects could potentially be quantified more precisely.

In addition to effects from the acquisition procedure, the low reproducibility of processing tools can also be a source of variability. Reproducibility quantifies how stable the results of a processing task or a sequence of tasks are when those are independently repeated across different computing platforms and at different times, a measure that a good processing pipeline should always maximize. Tractoflow (Theaud et al., 2020), a novel pipeline for DWI analysis of human subjects, was designed with this requirement in mind. Results displayed in Table 2 confirm good reproducibility of the computed DTI and fODF models, as well as of the T1w registration processes (using ANTs registration) and tissues partial volume fractions computation (using ANTs *Atropos*). Scores are slightly lower for metrics estimated on the DIAMOND model, and significantly lower for gamma distribution parameters (kappa, kappaAD, hei, and heiAD). This behavior is expected since the DIAMOND implementation included in the pipeline does not allow fixing the random number seed, which introduces variability in the outcomes of the algorithm. We would nonetheless advise against their usage in a statistical study since they are bound to introduce a level of variability in the analysis. Note that this does not affect the metrics calculated (MD, FA, max fFA, and fFW), since their computation in the current implementation of the pipeline only relies on the mean tensor estimated on each fascicle.

### 4.2. Pipeline implementation

While providing a highly reproducible processing chain, our pipeline implementation also offers a high level of adaptability to the parametrization of its input's acquisition sequences. While designing the collection of processing modules, we took great care to make their configuration resilient to different spatial (voxel size, number of slices, orientation of slice, and/or phase encoding) and orientational (number of b-values and/or gradient directions) configurations. This proved critical to adequately scale the parameter space of algorithms used by the pipeline, such as *Topup*, which rely on a definition of its deformation field given in millimeters in voxel space, and to allow disabling their execution when input requirements were unmet.

Nevertheless, we are aware that the default values we offer could vary as algorithms are updated and that some use-case could benefit from changing them. To that extent, we provided for most of them an additional configuration layer, presenting all possible parameters, to allow the end-user to finely adapt the pipeline to the specific study. We also allowed the user to disable all steps included in the pipeline, in case one desires only to compute models and measures and has already preprocessed the data, or to speed up computation time by disabling steps deemed unnecessary after thorough quality control. Still, for processing raw data, we do not recommend skipping any preprocessing steps.

Furthermore, our implementation is ready for new use-cases for which our pipeline does not provide adequate preprocessing and modeling steps. Using our modular design, a pipeline can easily be derived from ours, using new algorithms, workflows, and/or input conventions, by adding the necessary objects in their respective module and binding their dataflow correctly.

## 5. Limitations and future work

In the current version of *versaFlow*, we implemented a robust and efficient preprocessing chain of state-of-the-art algorithms to correct for well know artifacts affecting the MRI signal, such as background noise, Gibbs ringing, susceptibility distortions, motion, signal dropout, and intensity disparities within tissues. We are well aware that our pipeline is not universal and that some use-cases may require the execution of different algorithms than the one we considered. MRI preprocessing still needs to be thoroughly validated; the effect of applying specific techniques and algorithms, as well as their ordering relative to one another, requires quantification to attain an adequate level of consensus in the community. We designed our pipeline and its modular framework taking those considerations into account, to ease the process of swapping and upgrading its different steps, and to fit specific study cases. We also plan to release in the near future a version of the pipeline integrating alternative denoising techniques that are popular in the diffusion MRI research community. These denoising techniques will be selected according to recommendations from the *International Society for Magnetic Resonance in Medicine* (ISMRM) *Diffusion Study Group* (DSG) consensus effort. Inasmuch, we intend to include brain extraction workflows, for both anatomical and diffusion-weighted images, adapted to the morphology of input subjects. Good solutions have been developed that display good performance on human subjects. However, their extension to other primates and small animals is yet to be done; using them in the current version could lead to poor segmentation. As the field continues to evolve and those issues are addressed, we will consider the addition of those solutions in our pipeline. The same goes for harmonization techniques, which we consider adding to the pipeline in the near future, once their capabilities in reducing variability in DWI data while preserving intra-subject intrinsic variability and inter-subject variability have been thoroughly quantified.

Moreover, for our implementation, we chose a limited subset of 3 local models to represent the diffusion process in DW images. More algorithms, especially in the category of multi-tensor models, could be considered in the future, such as ball and stick (Behrens et al., 2003, 2007; Jbabdi et al., 2012), ball and zeppelin (Sotiropoulos et al., 2016), bedpostx (Jbabdi et al., 2012), and NODDI (Zhang et al., 2012). The inclusion of *Diffusion Kurtosis Imaging* (DKI) (Jensen et al., 2005) could also be considered. More general models of the *Ensemble Averaged Propagator* (EAP) could also be added, using, for example, the *Simple Harmonic Oscillator Based Reconstruction and Estimation* (3D-SHORE) or the *Mean Apparent Propagator* (MAP) (Özarslan et al., 2013), along with relevant measurements computed on them. Furthermore, we plan to release supplemental modules to compute tractograms upon the computed models the pipeline offers.

We also plan to extend our pipeline to support the processing of DWI data acquired *ex vivo* (Dyrby et al., 2011, 2018; Maffei et al., 2022; Yendiki et al., 2022; Schilling et al., 2023). Doing so requires solving a new set of challenges—response functions need to be recalibrated to account for changes in diffusivity incurred by tissue fixation and algorithms employing approaches based on templates acquired *in vivo* to be thoroughly tested and validated, to name a few. However, this would mean *in vivo* and *ex vivo* data from a given subject could potentially be processed using a common toolchain, guaranteeing minimal effects from the processing pipeline on further comparative analyses between the images—albeit configuration would differ slightly. Furthermore, we intend to add support for other species. The pipeline proposed was heavily inspired by similar processing chains implemented for human DW images and we think it would be a simple task to make it capable of processing human data. Support for other primates species, such as the marmoset, and for rodents is also in the works.

We also intend to address better computing resources management. The Nextflow scripting language offers extensive capabilities to define per-process resource prerequisites. Using them, we were able to define clearly the number of CPU cores and the need for hardware accelerators (GPU). However, we do not yet control for requirements for RAM of each individual process. The lack of specification for memory usage of multiple algorithms we used, combined with the difficulty of obtaining the actual memory space of compressed NIFTI images using the Nextflow language makes it difficult to define a clear requirement to respect at execution time. Specifying an overestimated precondition on memory needs could lead to a fewer number of processes executed at the same time, which would impede the parallelization capabilities of the pipeline and result in a waste of computing resources. We are aware that this behavior could lead to execution problems, more so when processing high-resolution images that span multiple gigabytes on disk. To mitigate the effects of this limitation, we opted for a retry strategy for processes failing for that reason, combined with a restriction on the number of parallel executions of memory-hungry processes.

## 6. Conclusion

In this study, we created a reliable and reproducible pipeline, *versaFlow*, capable of tackling the task of preprocessing and computing diffusion models on multi-resolution diffusion MRI data. We also presented our modular library of Nextflow processes and workflows using state-of-the-art and cutting-edge DW image processing technologies, designed to be easily upgradable and adaptable. We used our pipeline to analyze the variability of data from 3 sites (32 subjects) included in the PRIME-DE database presenting good quality DWI data. We showed that even if promising, that data exhibited a great level of variability and its usage should be done with care to prevent instilling uncertainty in statistical analyses. This is a good example of the effects of the lack of consensus in the diffusion MRI field when it comes to specifying guidelines for acquisition procedures. The hurdle of defining a gold standard in DWI makes this problem even more difficult to overpass, but a level of standardization and harmonization must be attained to reduce variability in imaging among subjects, scanners, and sites as much as possible. Without it, combined efforts like the PRIME-DE become less relevant for the study of the brain and its properties. It is through conjoint endeavors such as the consensus effort from the ISMRM diffusion study group and others, and by the development of reproducible, robust, and maintainable software, that we as a community will make diffusion MRI a reliable modality.

## Data availability statement

The datasets presented in this study can be found in online repositories. The names of the repository/repositories and accession number(s) can be found at: https://doi.org/10.5281/zenodo.5719453.

## Ethics statement

The data used in our study comes for a community driven database named PRIME-DE of already reviewed and approved data.

## Author contributions

AV, AS, and MD designed the project and the study. AS and MD funded the project. AV and MD designed the pipeline and the validation of the results. AV developed the pipeline, processed and analyzed the data, and wrote the article. MD, AS, and ZH reviewed the article. All authors approved the submitted version.
